# Development of a Novel Biomarker Platform for Profiling Key Protein–Protein Interactions to Predict the Efficacy of BH3-Mimetic Drugs

**DOI:** 10.3390/cancers17111852

**Published:** 2025-05-31

**Authors:** Andrew J. Kinloch, Faiyaz Rahman, Bahriye Karakas, Muhammad Shahid, Bora Lim, Stephanie J. Bouley, James A. Walker, Erinna F. Lee, Walter D. Fairlie, Kevin R. Kelly, Michael H. Cardone

**Affiliations:** 1 Eutropics Pharmaceuticals, Cambridge, MA 02138, USA; 2 MD Anderson Cancer Center, Houston, TX 77030, USA; 3 Center for Genomic Medicine, Massachusetts General Hospital, Boston, MA 02114, USA; 4 Cancer Program, Broad Institute of MIT and Harvard, Cambridge, MA 02142, USA; 5 Department of Neurology, Massachusetts General Hospital, Harvard Medical School, Boston, MA 02115, USA; 6 Olivia Newton-John Cancer Research Institute, Heidelberg, VIC 3084, Australia; 7 School of Cancer Medicine, La Trobe University, Bundoora, VIC 3086, Australia; 8 Department of Biochemistry and Chemistry, School of Agriculture, Biomedicine and Environment, La Trobe University, Bundoora, VIC 3086, Australia; 9 La Trobe Institute for Molecular Science, La Trobe University, Bundoora, VIC 3086, Australia; 10 Keck School of Medicine of USC, Los Angeles, CA 90033, USA

**Keywords:** apoptosis, BH3 domain, PRIMAB, AML, mitochondrial priming, BH3 mimetics, flow cytometry

## Abstract

One of the hallmarks of cancer cells is their failure to respond to the cellular mechanism of apoptosis. The B-cell lymphoma 2 (BCL-2) family of proteins regulate apoptosis. Their ability to do so can be measured using several methods that in turn anticipate the fate of the cancer cell in response to apoptosis-inducing treatment. These assays ultimately identify the readiness of the cancer cell to undergo apoptosis, which is referred to as the mitochondrial priming state. These metrics, however, have been challenging to implement in the clinic. **Methods**: Here, we describe a unique method that relies on a panel of novel conformation-specific antibodies (termed PRIMAB) that can directly measure the mitochondrial priming state. These reagents are highly specific for complexes of their corresponding pro-survival protein interactions with the pro-apoptotic protein BIM. These BIM-containing heterodimeric complexes have long been established as hallmarks of primed cancer cells. **Results**: Using clinically amenable assay formats, PRIMABs were shown to detect the presence of these anti-apoptotic–pro-apoptotic complexes and their disruption by BH3-mimetic drugs. Moreover, PRIMABs were able to detect a shift in priming status following BH3-mimetic treatment, a factor associated with resistance to these drugs. In a panel of AML patient samples, we report a wide range of priming levels for each PRIMAB complex, demonstrating the potential for heterogeneity in responses. We also show that PRIMABs could be predictive of outcomes for AML patients following cytarabine-based treatment. **Conclusions**: PRIMABs provide novel and useful tools for cancer research and for clinical implementation as reagents providing predictive tests for treatment response.

## Simple Summary

The process of developing improved treatments for cancer is increasingly dependent on accurate clinical tests, enabling clinicians to identify if a given drug will be effective in an individual cancer patient. Such tests improve the likelihood of a positive therapeutic response and avoid unnecessary adverse effects in patients. Eutropics’s predictive diagnostic assay platform has unique potential to provide such useful information, facilitating both improved drug development and successful treatment of cancer patients. This capability is derived from PRIMABs^TM^-DX, a panel of novel conformation-specific antibodies that measure the ability of a cancer cell to avoid treatment-mediated cell death induced by BH3 mimetics. We show here the development of the test platform and how it works in the context of acute myeloid leukemia (AML) patient biopsied samples.

## 1. Introduction

Diagnostic tools for cancer therapy that enable oncologists to inform patients about the likely benefits of their treatment versus toxicity empower patients to make evidence-based decisions about the management of their disease [1]. The occurrence of hallmark genetic mutations provides useful information for matching a targeting treatment with the small percentage of patients who have those mutations [2], but there is widespread uncertainty about the broad clinical value of these approaches [3,4]. Proteomic and functional assays provide another layer of utility, but they also have limitations. This has left an unmet need for broadly applicable predictive testing [5].

Most cancer treatments work through the activation of the cell suicide program of apoptosis. This process is tightly regulated by the BCL-2 family of proteins, the members of which either prevent or promote cell death, with the latter group including a subset of proteins referred to as BH3-only proteins characterized by their conserved BH3 binding domain [6]. The control of this event occurs by each subset of the BCL-2 family proteins antagonizing each other through protein–protein interactions (PPIs) [7]. These interactions involve a hydrophobic pocket on the anti-apoptotic BCL-2 members that act as a receptor for the BH3 domain of the pro-apoptotic proteins. Accordingly, the interactions between the BCL-2 family of pro- and anti-apoptotic proteins are crucial for determining cell fate, including drug-induced apoptosis [8,9]. The multidomain anti-apoptotic BCL-2 family proteins BCL-2, BCL-XL, BCL-W, MCL-1, and BFL-1 prevent apoptosis by counteracting BH3-only proteins such as BIM and BID that can directly activate BAX and BAK to cause mitochondrial outer membrane permeabilization (MOMP), a pivotal step in apoptosis [10,11]. Additional “sensitizer” BH3-only proteins, including BAD, HRK, NOXA, and others, are also sequestered and antagonized by anti-apoptotic BCL-2 family proteins [12].

Cancer cells can be characterized by their relative levels of pro- and anti-apoptotic proteins, which determine their propensity to undergo apoptosis. Cells that more readily die in response to particular stimuli are referred to as “primed”, as they have higher levels of the anti-apoptotic proteins’ hydrophobic pocket bound by pro-apoptotic BH3 domains [13]. Hence, the degree of priming of a tumor sample can be predictive of responses to anticancer drugs and measured using various assays [12,13,14,15]. Such measurements are important in predicting the potential for successful cancer treatment for a broad class of drugs that rely on the occurrence of MOMP [16,17,18,19]. Many of these therapies work upstream in the apoptotic pathways by upregulating BH3-only proteins that ultimately converge on the mitochondria. The BH3-mimetic class of therapies work by directly disrupting the BH3 domain-mediated anti-apoptotic–pro-apoptotic PPIs [20]. Among them is the FDA-approved Venetoclax, a BCL-2:BIM complex disrupter. This drug is successfully used in the treatment of various blood malignancies including acute myeloid leukemia (AML), where it is administered in combination with hypomethylating agents (HMAs) or low-dose cytarabine (LDAC). Venetoclax has become the standard of care for AML patients who are 75 or over in addition to specific relapsed groups [21]. Venetoclax is also used in chronic lymphocytic leukemia (CLL), and testing is underway in other cancers, including multiple myeloma (MM) [22]. Several next-generation BCL-2:BIM-disrupting BH3-mimetics, including Sonrotoclax and Lisaftoclax, are also under development for clinical use. Additional BH3-mimetics that co-target BCL-2 and BCL-XL, MCL-1, or BCL-XL are in various stages of clinical development [23,24]. Because each of the anti-apoptotic proteins function as a driver of tumorigenesis and resistance to treatment or relapse, a survey of their involvement is important in determining the extent of an individual cancer patient’s oncodependence on these respective proteins. Moreover, identifying the specific protein complexes involved in survival reveals the cancer cell vulnerabilities and, therefore, guides the use of an appropriate class of BH3-mimetics whether alone or as part of a combination treatment.

Currently, there are no universally accepted laboratory markers to precisely predict Venetoclax sensitivity or resistance in AML. Anecdotally, high levels of BCL-2 have been associated with better response; in comparison, low gene expression of BCL2A1 and MCL-1, as well as other (non-BCL-2 family) proteins such as CD14 and CLEC7A (CD369), have correlated with Venetoclax sensitivity [25]. Genomic mutational analysis performs well in identifying risk categorization of patients and, in a small number of reports, has predicted targeted therapeutic success in mutational subgroups, representing a small percentage of cancer patients [26]. AML patients with *IDH1/2*, *FLT3, WT1,* and *TET2* mutations are associated with greater sensitivity and higher response rates to Venetoclax [27]. A more comprehensive approach in which molecular biomarkers are combined with proteomic and functional metrics could yield more robust correlations and response predictions. Nongenetic approaches are also being explored for predictive utility. For instance, BCL-2 family protein measurement, as individual proteins or in combination with other readouts, shows some promise for predicting treatment response in blood and solid tumors [15,28,29].

Measurement of BCL-2 anti-apoptotic–pro-apoptotic protein complexes as surrogate functional readouts for BCL-2 family protein function has been considered a promising predictive metric, as it has been used widely since the early days of BCL-2 biology research. For instance, coimmunoprecipitation of complexed proteins followed by Western blotting or ELISA-based detection has enabled measurement and informed the significance of these PPIs. Relative levels of a panel of BCL-2 anti-apoptotic–pro-apoptotic protein complexes predicted sensitivity to Venetoclax and other BH3 mimetics in a pre- and post-treatment comparison [13,14,15]. This methodology, however, is extraordinarily complex and has been challenging to adapt for use as commercial clinical tests.

Another surrogate functional method of measuring priming PPIs pre- and post-treatment is BH3 profiling, which has been investigated for use as a functional predictor of cancer chemotherapy response. BH3 profiling requires treating permeabilized cancer cells with peptides composed of the BH3 binding domains or BH3-mimetic small molecules and subsequently measuring the resulting onset of MOMP [12,30,31,32,33]. In principle, the response to the peptide can reveal the cell priming state and oncodependence on the respective pro-apoptotic–anti-apoptotic complex. Selection of the best treatment option that would target these dependencies could then be made. In hundreds of studies, BH3 profiling has been valuable in measuring BCL-2 protein priming. However, reluctance to adopt it as a routine clinical test is likely due to the fastidious nature of the assay, including the critical need for cell viability and the consistent induction of partial cell permeabilization using detergent.

To address the technical limitations of coimmunoprecipitation, BH3 profiling, and other functional assays [34], we developed a unique panel of mitochondrial priming state-detecting monoclonal antibodies referred to henceforth as PRIMABs. Unlike BH3 profiling, which is an indirect measure and reliant on viable cells and standardized permeabilized states, PRIMABs measure tumor cell priming directly in situ, providing quantitative and spatial information on fixed biopsied tissues. Herein, we describe the characterization of PRIMABs, demonstrate their potential utility in guiding treatment in liquid and solid tumors, and illustrate the feasibility of our novel PRIMABs-DX platform for predicting therapeutic response in AML patients. Further, we demonstrate how this platform yields a clinically translatable pharmacodynamic (PD) biomarker that directly measures the key BCL-2:BIM PPIs.

## 2. Materials and Methods

### 2.1. Monoclonal Ab (mAb) Generation

Immunogens for mouse immunization and substrates for downstream hybridoma selection were prepared for the generation of mAbs to their corresponding heterodimer complexes using the two different strategies outlined below.

i. Anti-MCL-1:BIM and Anti-BCL-XL:BIM

Human MCL-1-glutathione-S-transferase (GST) and BCL-XL-GST fusion proteins were cloned into the pGEX 4T-1 expression plasmid (available from Addgene, Watertown, MA, USA). Proteins were expressed in BL21 (DE3) *E.coli* and purified using Amersham HiTrap glutathione columns on an AKTA-FPLC as previously described [16].

Using a proprietary process, BCL-XL:BIM heterodimer was prepared by covalently linking BCL-XL to the BIM-BH3 domain peptide (Supplemental Illustration S1). The peptide was selected following an antecedent screen in which a series of BIM-BH3 domain peptides were prepared (C-AHA-MRPEIWIAQELRRIGDEFNA-[NH2]) with 4-benzoyl phenylalanine (BPA) residues. These modified peptides were synthesized at Anaspec (Fremont, CA, USA) and used to replace the sterically similar aromatic amino acids in the peptide one by one (Supplemental Figure S2). The modified peptides were then tested for their BCL-XL binding affinity using fluorescence polarization assays as described previously [16]. The BPA-modified peptide with the most similar binding affinity to the wild-type BIM BH3 peptide (4 nM) was chosen for covalent linking. This was performed by adding a 2-fold molar excess of BPA-BIM-BH3 to BCL-XL-GST and exposing the complex to UV light for 1 h [35]. After reaction completion, the unbound BCL-XL-GST was removed by purification using a biotinylated-BIM BH3-streptavidin-bead column (Arco Biosystems, Newark, DE, USA). The flow-through was collected and prepared for mouse immunization. An analogous method was used to generate MCL-1:BIM complexes. PRIMABs were generated following immunization with the immunogen in an emulsion mix with complete Freund’s adjuvant into DiversimAb mice (Abveris Inc., Quincy, MA, USA). Hybridoma clones were selected for the following binding properties by ELISA: (i) BCL-XL:BIM and MCL-1:BIM specificity; (ii) absence of binding to monomeric forms of BCL-XL, MCL-1, and BCL-2 or BIM.

ii. Anti-BCL-2:BIM

In contrast to the BCL-XL and MCL-1 immunogens, the BCL-2:BIM complex immunogen was a proprietary recombinant antigen composed of the BH3 domain-containing segment of BIM tethered with a glycine linker to GST-BCL-2. DiversimAb mice (Abveris Inc.) were immunized with the BCL-2:BIM immunogen at Abveris (now TWIST, South San Francisco, CA USA). Hybridoma cell lines secreting antibodies that specifically bound BCL-2:BIM complex, but not the corresponding monomers, other BCL-2 family member monomers, or BCL-2 complexed with BID, BAD, or BAX, moved forward for flow cytometry, immunofluorescence (IF), and immunohistochemistry (IHC) testing.

Occupation of the BH3 binding hydrophobic pocket in all immunogens was confirmed using fluorescence polarization following methods described previously [16].

### 2.2. ELISA for Heterodimer Specificity of PRIMABs

For the negative screen, glutathione-coated ELISA plates (Thermo Scientific Pierce, Cat# 15140, Waltham, MA, USA) were coated with 100 µL/well of the following individual anti-apoptotic BCL-2 family proteins: GST-BCL-XL (1 µg/mL), GST-BCL-2 (1 µg/mL), or GST-MCL-1 (1 µg/mL) diluted in PBS. Background control wells were rinsed with 100 µL/well PBS. Unbound antigen was washed out twice with 300 µL/well PBS + 0.1% Tween-20. Wells were then blocked with 300 μL PBS + 3% (*w*/*v*) BSA for 1 h. To construct the PRIMAB target antigen for the positive screen, the anti-apoptotic BCL-2 family members of the respective protein complexes were first coated on the wells as above. The plate-bound anti-apoptotic proteins were subsequently complexed with full-length recombinant BIM protein or BIM BH3-domain-containing peptides at 1 µM (unless otherwise stated) in 100 µL/well for 2 h at room temperature with shaking. After two washes with 300 µL/well PBS + 0.1% Tween-20, 100 µL/well of each hybridoma supernatant-derived candidate PRIMAB clone (at the indicated concentrations) was added, and the plate was incubated for one hour. Unbound mAb was washed off with three 300 µL/well washes of PBS + 0.1% Tween-20. Bound heterodimer-specific mouse IgG was then detected with 100 µL/well of HRP conjugated donkey anti-murine IgG (Jackson ImmunoResearch Labs, West Grove, PA, USA) diluted 1:3000 in PBS + 3% (*w*/*v*) BSA. Wells were washed three times with PBS + 0.1% Tween 20, then treated with 100 µL/well of 1-step Ultra TMB substrate (Thermo Fisher Scientific, Cat#34028, Waltham, MA, USA). Once sufficient blue intensity was observed, the reaction was stopped with the addition of 50 µL/well 0.1 M H_2_SO_4_. Optical densities for wells were recorded at 450 nm on a Tecan Infinite F Plex plate reader (Tecan, Männedorf, Switzerland). To establish the specificity of the PRIMABs, protein complexes coated on wells as above were treated with a 10-fold dilution series of BH3-mimetics (ABT-199 to target BCL-2, A1331852 and A1155463 to target BCL-XL, and ABT-263 to target BCL-XL+BCL-2). All treatments with BH3 mimetics were carried out for 1 h at room temperature prior to analysis by ELISA as described above.

### 2.3. PRIMAB Assessment of Intracellular BCL-2 Family PPIs Using Flow

H929, U266B1, U2932, MCF7, and HT229 cell lines were selected based on historic demonstrations of their dependences on corresponding BCL-2-family members [14,16,29]. Except for MCF7, HT229, and HCC1937 (gifts from Dr. Bora Limb, MD Anderson Cancer Research Center, Houston, TX, USA), all cell lines were purchased from the American Type Culture Collection (ATCC, Manassas, VA, USA; Supplemental Table S1). Cell lines not recently purchased were tested for cell line authenticity by measuring short tandem repeats by STR profiling (Wi Cell, Madison, WI, USA). Cell lines were routinely tested for mycoplasma using a MycoAlert Mycoplasma Detection Kit (Lonza, Basel, Switzerland). All cell lines were cultured as per manufacturers’ guidelines. H929, U266B1, U2932, and HCC1937 were grown in RPMI-1640 (Fisher Scientific, Waltham, MA, USA) with 10% fetal bovine serum (FBS). MCF7, MEF, and HT229 were grown in DMEM with 2 mM L-glutamine (Fisher Scientific, Waltham, MA, USA) and 10% FBS. All media were changed to containing 5% FBS during treatments.

Suspension cell lines were plated in 96-well plates (Greiner Bio-One, Monroe, NC, USA) at 30,000 cells/well and treated at the indicated times at 0.5% CO2/37 °C with BH3-mimetics targeting the critical BCL-2 family members BCL-2 (ABT-199/Venetoclax, 2 µM for 3 h), MCL-1 (AZD5991, 1 µM for 6 h), or BCL-XL (A1155463, 3 µM for 8 h), or with an equivalent volume of DMSO as a control (Figure 2A). Cells were then rinsed with PBS, fixed with 4% (*v*/*v*) paraformaldehyde, permeabilized with 0.1% (*v*/*v*) Triton-X 100, and blocked with 1% (*w*/*v*) BSA. Cells were then stained with PRIMABs specific for BCL-2:BIM (HSB2B), MCL-1:BIM (HSMCB), or BCL-XL:BIM (HSBXB) (see Supplemental Table S1 for details). BCL-2:BIM complexes were detected with APC-labeled anti-mouse secondary antibodies (Table 1). To measure total (complexed and noncomplexed) BCL-2, MCL-1, BCL-XL, or BIM, the same protocol was followed except using commercially available fluorescent labeled rabbit monoclonal antibodies against each target BCL-2 protein (Table 1). Fluorescent signals were then read on a Canto 2 flow cytometer (BD Biosciences, Franklin Lakes, NJ, USA) as mean fluorescence intensities (MFI) in the selected gates. FACSDiva v6.1 and FlowJo software v10.0.8 (BD Biosciences) were used to collect and analyze data. For analysis, both the MFI and the “priming ratio” (PRIMAB MFI divided by constituent anti-apoptotic protein MFI (i.e., [HSB2B/total BCL-2], [HSMCB /total MCL-1], [HSBXB/total BCL-XL])) were used. Based on these data, we established the range of values representative of HIGH (e.g., from H929 cells known to be highly primed cells for MCL-1 [16]) and LOW (e.g., from BIM KO cell lines) signals. The acceptance criterion was <10% CV for HIGH, and <30% CV for LOW.

The selective association of PRIMAB signals with BH3-mimetic sensitivities (priming), were demonstrated further in the earlier stage of apoptosis. For this, the IC50s for each of the BH3 mimetics BCL-2 (ABT-199/Venetoclax), MCL-1 (AZD5991), and BCL-XL (A1155463) in cell lines H929, U266B1, and U2932 were determined. Cells were grown as above and treated with each of the BH3 mimetics titrated from 50 nM to 5 µM by 2-fold serial dilution and treated with these for 24 h. The Presto Blue (Thermo Fisher Scientific, Waltham, MA, USA) signal was then read on a Tecan Infinite plate reader according to the manufacturer’s specification to determine cell death. The IC50s for each of the cell lines were determined using GraphPad Prism v8.3 (nonlinear regression and dose–response curve fitting) and plotted against the % fold change of PRIMAB reading from baseline and at 1 h of treatment with each of the BH3 mimetics at 1 µM. Baseline readings were set at 1, and the post treatment % fold change in the BCL-2 BIM complex measurements = (Fold change − 1) × 100.

### 2.4. BIM siRNA and BCL-XL siRNA Knockdown

To further confirm the requirement for BIM binding for the HSMCB binding, MCL-1:BIM-positive MCF7 breast cancer cells were transfected in 6-well plates with ON-TARGETplus SMARTpool siRNA against BIM (*BCL2L11*; Dharmacon, Cat#SO-2704885G, Layfayette, CO, USA) using the DharmaFECT transfection reagent (Horizon Discovery, Cat#T-2001-01, Waterbeach, UK) in serum-free medium. BIM siRNAs: GCGGAGAAAUCAAGUUUAA, GGAAGUUUGUUGUGAAUGU, UGAGUCAGAACAAGAGUUA, and UCUUACGACUGUUACGUUA. The transfection cocktail (5 µM siRNA) was mixed and applied to cells following a 20 min incubation at room temperature at a final concentration of 25 nM using antibiotic-free transfection medium (see Supplemental Table S2). Cells were incubated at 37 °C in 5% CO2 for 48 h and analyzed for expression of BIM protein using phycoerythrin conjugated rabbit anti-BIM (Cell Signaling Technologies, Danvers, MA, USA) and complexes of BIM with BCL-2 family members using HSMCB, HSB2B, and HSBXB.

Similar transfection was carried out using siRNA against BCL-XL (ON-TARGETplus Human *BCL2L1* siRNA SMARTpool, Dharmacon, (Lafayette, CO, USA) Cat#SO-2702960G) GGACAGCAUAUCAGAGCUU; GAAAUGACCAGACACUGAC; CCUACAAGCUUUCCCAGAA; UUAGUGAUGUGGAAGAGAA.

### 2.5. BIM CRISPR Knockout MCF7 Cell Line Preparation

MCF7 cells (1 × 10^6^) were transfected with 1 µg of a 12:1 DNA ratio of the pCMV-T7-(FZ) SpCas9-BPNLS(SV40)-3xFLAG-P2A-Puro-R plasmid (modified from RTW3027, Addgene #139987) and a plasmid (derived from BPK1520, Addgene #65777) with a sgRNA targeting exon 5 of *BIM* using Polyethylenimine (PEI) at a 4:1 ratio. Following transfection, selection was performed using 1 µg/mL puromycin for 24 h. After recovery for 4 days, cells were plated for clonal selection and expansion. Clones were initially screened by PCR and Sanger sequencing, followed by next-generation sequencing (NGS) to confirm successful disruption of all four alleles of *BIM*. sgRNA: 5′-ATGGATCGCCCAAGAGTTG-3′. PCR primers: FOR: 5′-GTGAGATGGGCTTGCCTCTA-3′, REV: 5′-TGGGAAAGGAGAAACAATGAA-3′. NGS primers: FOR: 5′-GTGAGATGGGCTTGCCTCTA-3′, REV: 5′-AACATCATTACCCTCCTTGCAT-3′. MCF7 WT and *BIM* KO cell lines were also characterized by Western blotting using lysates prepared in RIPA buffer supplemented with protease inhibitors (Complete Protease Inhibitor Cocktail, Sigma, St. Louis, MO, USA). Lysates were analyzed on NuPAGE 12% Bis-Tris gels (Thermo Fisher Scientific, Waltham, MA, USA) before transferring to nitrocellulose membranes and processing using the Odyssey CLx protocol (LI-COR, Lincoln, NE, USA). Antibodies: anti-human C-terminal BIM (goat, Bio-RAD, Hercules, CA, USA, AHP2074; 1:1000), anti-human N-terminal BIM (C34C5) (rabbit, Cell Signaling, #2933; 1:1000), and anti-b-tubulin (mouse, Developmental Studies Hybridoma Bank E7, Iowa City, IA USA, 1:10,000). Secondary antibodies used were anti-goat Alexa Fluor Plus 800 (donkey, Invitrogen, Waltham, MA, USA, A32930, 1:10,000), anti-rabbit IgG (H+L) DyLight 800 (goat, Thermo Fisher Scientific, Waltham, MA, USA, 35571, 1:10,000), and anti-mouse Alexa Fluor Plus 800 (goat, Invitrogen, A32730, 1:10,000).

### 2.6. Assessment of PRIMABs as Biomarkers in Immunohistochemistry (IHC) on Cell Block Samples of Tissue Microarrays (TMAs)

Tissue microarrays (TMAs) were made from blocks of U2932, H929, and U266B1 cell lines treated or untreated with ABT-199, AZD5591, or A1155463, respectively, as described in Supplemental Table S3. Following treatments, 35 × 10^6^ cells per sample were pelleted, washed, and fixed in 4% (*v*/*v*) formalin and sent to Alamak Biosciences (Beverly, MA, USA), where they were transferred as concentrated specimens to a flat-bottom glass tube. As a marker for cell identification, 0.01% India ink was added; cells were then mixed with molten HistoGel (Thermo Fisher Scientific, Waltham, MA, USA) to position cells in the cutting surface, and cell blocks were cut and processed into TMAs.

### 2.7. Immunohistochemistry on Formalin-Fixed and Paraffin-Embedded (FFPE) Tissue/Cell Substrates

Slides coated with FFPE substrates were deparaffinized by incubating for 30 min at 65 °C in a drying oven. Samples were then rehydrated sequentially in (i) xylene, 3 × 10 min; (ii) 100% ethanol, 2 × 5 min; (iii) 95% ethanol 1 × 5 min; (iv) 70% ethanol, 1 × 5 min; (v) dH_2_O, 1 × 5 min. Slides were equilibrated in PBS for 5 min. Antigen retrieval at pH 9.0 was performed for 20 min at 96 °C followed by an additional 60 min incubation at 4 °C. Antigen retrieval buffer (Vector Laboratories catalog #H-3301-250) was rinsed off with three 5 min PBS washes. Hydrophobic barriers around the substrate were marked with a PAP pen, endogenous peroxidase activity was blocked with BLOXALL (Vector Laboratories) for 10 min, and slides were washed for 5 min in PBS. Substrates were permeabilized and incubated in blocking buffer (PBS + 5% normal donkey serum + 0.05% (*v*/*v*) Triton-X100) for one hour. Slides were then stained overnight with primary antibody (diluted in blocking buffer) at 4 °C. Slides were washed in PBS + 0.01% (*v*/*v*) Tween-20 (3 × 5 min), and bound antibody was detected with HRP-conjugated secondary antibody (diluted in blocking buffer at 1:1000) for 60 min at room temperature. Secondary antibody was washed out as above with three washes of PBS + 0.01% (*v*/*v*) Tween-20. Slides were incubated in impact DAB (Vector Laboratories) brown chromogen for 5 min followed by a 5 min washout with running water. Counterstaining of nuclei was performed for 2 min with Hematoxylin QS (Vector Laboratories). Samples were washed again for 5 min in running water, then dehydrated using 10 s incubations of the rehydration buffers in the reverse order; slides were left to dry prior to mounting. Images were resolved and analyzed using HALO (Indica Labs, Albuquerque, NM, USA) or Image Quant (Cytiva, Marlborough, MA, USA). Post-fixation antigen retrieval details are described in Supplemental Table S3.

Tissues were scored for extent and intensity of staining using the HALO image analysis software v3.2, which generates a “mean cytoplasmic optical density” (MCOD) for cells per field of view, enabling the degree of priming to be established by the ratio of the MCOD for each of the PRIMABs stained to the MCOD for slides stained with antibodies to the corresponding unbound pro-survival proteins and BIM. A “combined” priming score was generated by summing the scores for each of the PRIMABs.

### 2.8. IF Imaging of BCL-2 Family PPIs in Breast Cancer Cell Line Using PRIMABs

HCC1937 breast cancer cells were grown in DMEM (Fisher Scientific, Waltham, MA, USA) with 10% Fetal Calf Serum (FCS) plus 2 mM L-Glutamine in 8-well glass chamber slides (EZ Slide, Millipore, Burlington, MA, USA) until near confluence. Cells were treated with 50 nM A1331852 plus 50 µM Z-VAD-FMK (benzyloxycarbonyl-Val-Ala-Asp-fluoromethylketone) (BD Biosciences, San Jose, CA, USA) for 24 h or left untreated and incubated under standard tissue culture conditions. Following treatment, cells were rinsed and fixed with 4% PFA in PBS for 10 min at room temperature and permeabilized with 0.05% Triton-X100 in PBS plus 0.1% (*w*/*v*) BSA. Cells were stained with 1 µg/mL rabbit polyclonal anti-BCL-XL and 1 µg/mL mouse monoclonal HSBXB PRIMAB in blocking buffer overnight at 4 °C. Unbound antibody was washed away with three PBS washes. Bound HSBXB was detected with anti-mouse-Alexa-488 (Invitrogen, Waltham, MA, USA). Bound BCL-XL was detected with anti-rabbit-IgG-Alexa-568 (Invitrogen) following a one-hour room-temperature incubation. Samples were rinsed and mounted with Vectashield (Vector Laboratories, Newark, CA USA). Image acquisition was performed using a Zeiss LSM 880 with Airyscan confocal microscope (Zeiss, Oberkochen, Germany) and analyses performed with ZEN imaging software v2.3 at the Harvard University Center for Bioimaging.

### 2.9. PRIMAB (HSMCB) Detection of CDK9 Disruption of MCL-1:BIM Complexes in Myeloma Cells

H929 cells were grown in RPMI-1640 (Fisher Scientific, Waltham, MA, USA) with 10% FBS at 30,000 per well (96 well plates) as above. Growth phase cells were treated with the CDK-9-inhibiting compound Enitociclib (Medchem Express, Monmouth Junction, NJ, USA). Treatments were at 0.1 µM, 1 µM, and 10 µM for 3 h, 6 h, and 16 h. Following treatment cells were rinsed, fixed, and stained with HSMCB and rabbit anti-MCL-1 conjugated to phycoerythrin-CY 7 (Cell Signaling Technologies, Essex, MA, USA) as described above. HSMCB signal was detected with anti-mouse secondary (LifeTechnologies, Carlsbad, CA, USA). Flow cytometry analysis was performed on a FACSCanto II, and signals were analyzed using FACSDiva (BD Biosciences).

### 2.10. PRIMAB Correlation with IC_50_s EGFR Inhibitors in Myeloid Cells

PRIMAB measurements of BCL-2:BIM, BCL-XL:BIM, and MCL-1:BIM complexes in the human leukemia cell lines KG1a, MV411, EOL-1, SKM-1, F36-P. MOLM-13, NB4, HEL 92.1.7, and SKM-1 were obtained. Cells were thawed, rinsed, fixed, permeabilized, and stained with the PRIMABs as described above. The IC_50_s for the EGFR inhibitors Cetuximab, Osimertinib, and Dacomitinib were obtained from the Broad Institute Cancer Cell Encyclopedia.

### 2.11. PRIMAB Technical Performance and Signal Range in AML Patient Samples

Technical assessment of each of the PRIMABs was conducted using human leukemia and myeloma cell lines. Staining and flow analysis was performed as described above. Flow cytometry runs were performed on cell lines (H929, U266B1, U2932, THP1, ML2, KG1a, MV411, HAL929.1.7, EOL-1, SKM-1, F-36P, MOLM-13, NB4, and RS4-1) with the same operator testing on different days (intraday) and with different operators testing on the same day (intraoperator). Cells grown in suspension were plated at 30,000 cells/well in a 96-well plate (Greiner Bio-One, Monroe, NC, USA). Cells were rinsed with PBS, fixed with 4% (*v*/*v*) paraformaldehyde, permeabilized with 0.1% (*v*/*v*) Triton-X 100, and blocked with 1% (*w*/*v*) BSA. Cells were then stained with PRIMABs specific for BCL-2:BIM (HSB2B), MCL-1:BIM (HSMCB), or BCL-XL:BIM (HSBXB) (see above for assay details). Cell lines or biopsied cells could be viably frozen, stored, and then fixed or fixed at time of harvesting. We observed no diminution in PRIMAB signal after 4 years of storage in liquid nitrogen. Fixed samples could be stored for up to 4 weeks before analyzing.

### 2.12. Assessing Signal Range in Naive AML Samples

AML patient bone marrow peripheral blood biopsy samples purchased from ReproCell Global (Beltsville, MD, USA) were used to further establish the operating range of PRIMAB signals. Testing was performed using flow cytometry analysis of PRIMAB signals as described below.

### 2.13. Measurement of BCL-2 Complexes in Biopsied AML Patient Samples Using PRIMABs

Newly diagnosed AML patient samples were obtained prior to induction chemotherapy administration at Cardiff University. Specimens were acquired during routine diagnostic assessments in accordance with the regulations and protocols of AML 19 Trial: Patient Information Sheet 9 (Ref: ISRCTN) approved by the investigation review board of Cardiff University. Informed consent was obtained in accordance with the Declaration of Helsinki.

Patients in the study ranged in age from 24 to 75. All were treated with 7 + 3 cytarabine + anthracycline [*n* = 21]. Biopsies were from peripheral blood (PB) in patients with whole blood cell counts > 10,000 and > 20% blasts in PB, or from bone marrow (BM). Leucocytes were isolated by Ficoll separation. Samples were either fixed and stored appropriately or viably frozen as described in [29] and stored for use with the PRIMABs-DX platform. When there were sufficient viable cells (≥4 million leukocytes), BH3 profiling was also performed.

Specimens (*n* = 21 patients) consisted of 7 BM and 14 PB samples. AML specimens were washed after thawing and suspended in 1% FBS + 2 mM EDTA-PBS for staining with primary antibodies CD45-V450 (BD Biosciences, San Jose, CA, USA). When possible, patient samples (Ficoll-purified PB or BM) were fixed and stained for PRIMABs and constituent monomer proteins as above with the additional steps of immunophenotyping for the AML Blast cell population for analysis.

Purified leucocytes were fixed with 4% paraformaldehyde, permeabilized with 0.1% (*v*/*v*) Triton-X 100, blocked, and stained with PRIMABs specific for BCL-2:BIM (HSB2B), MCL-1:BIM (HSMCB), or BCL-XL:BIM (HSBXB) individually and resolved with labeled anti-mouse secondary antibodies. To measure total BCL-2, MCL-1, BCL-XL, and BIM, rabbit monoclonal antibodies against each protein were used. Each had a nonoverlapping fluorescent label, and all were read together in a single well on a FACS Canto 2 flow cytometer. FACSDiva v6.1 and FlowJo software v10.0.08 (BD Biosciences, San Jose, CA, USA) was used to collect and analyze data. For analysis, both the MFI and the “priming ratio” (PRIMAB MFI divided by constituent anti-apoptotic protein MFI ([HSB2B/total BCL-2]; [HSMCB/total MCL-1]; [HSBXB/total BCLX-L]) were used. Acceptance for upper and lower limits were as described above.

Event-free survival (EFS) was established as the time from the start of treatment to the occurrence of primary refractory disease, relapse, or death. Relapse was determined to occur when more than 5% blasts in the bone marrow or blasts in the peripheral blood occurred in a patient formerly in complete response (CR). Primary refractory disease (no response, NR) denoted residual leukemia after 2 cycles of induction chemotherapy. For statistical analyses, CR denoted patients who exhibited response with or without subsequent relapse, and NR showed primary refractory disease. Cell lines with known BCL-2 family dependencies and treatments were used to define the range (LOW–HIGH) of responses. The analytical accuracy was determined by comparing the results obtained from flow cytometry for each PRIMAB and monoclonal antibody against each of the constituent proteins in the complex (BCL-2, BCL-XL, MCL-1, and BIM).

### 2.14. BH3 Profiling of AML Patient Samples

Assays were performed in flow cytometry format as previously described [33]. Viable frozen AML specimens were washed after thawing and suspended in 1% FBS + 2 mM EDTA-PBS for staining with primary antibodies CD45-V450 (BD Biosciences), CD3-biotin (BD Biosciences), and CD20-biotin (eBiosciences, San Diego, CA, USA) and secondary antibody streptavidin-APC (BD Biosciences). Specimens were then permeabilized with digitonin (Sigma-Aldrich, St Louis, MO, USA) and incubated with JC-1 mitochondrial dye (Enzo Life Sciences) and either BH3-domain-containing peptides, DMSO, or carbonyl cyanide *m*-chlorophenyl hydrazone (Cayman Chemicals, Ann Arbor, MI USA). Samples were run in duplicate and analyzed on a FACSCanto II using the FACSDiva software v6.1 (BD Biosciences). The blast population was identified as CD45 dim, CD3 negative, and CD20 negative. Intensely stained CD45 cells (mature lymphocytes) were excluded from analyses as described previously [33]. The quantifiable propensity of a pro-apoptotic peptide (BH3 only) to induce mitochondrial depolarization relative to an uncoupling reagent control is referred to as percent priming. For the blast population, this was calculated using the median signal intensity of the PE channel normalized for DMSO as background (negative control), with CCCP serving as 100% priming (positive control). For calculating the % priming, the following formula was utilized:

% Priming = 1 − ([peptide − CCCP]/[DMSO − CCCP])

### 2.15. BH3 Profiling of WT and BIM-KO MCF7 Breast Cancer Cell Lines

BH3 profiling, a method that involves the perturbation of cells with specific BH3 domain peptides followed by measurements of their response in terms of induction of apoptosis, was performed on MCF7-WT and MCF7-BIM-KO cell lines in a 96-well-plate-based assay as described previously [5,6]. Briefly, cells were permeabilized with digitonin (Sigma-Aldrich, St. Louis, MO, USA), incubated with JC-1 (Enzo Life Sciences, Farmingdale, NY USA), and exposed to various BH3 peptides (100 µM BIM, 0.1 µM BIM, 100 µM PUMA, 100 µM NOXA, 50 µM NOXA,100 µM BAD, 100 µM L HRK, 100 µM BID, 50 µM MS1, or 10 µM MS1) (New England Peptides/Biosynth, Boston, MA, USA) at room temperature. CCCP (10 µMol/L) was used to completely depolarize mitochondria and provide a positive control. An Infinite plate reader (Tecan) was used to detect shift fluorescence from JC-1 dye aggregation dispersal as a surrogate for depolarization. The area under the curve for each condition was measured, and the proportion of depolarization was calculated using the following equation:

% Priming = 1 − ([peptide − CCCP]/[DMSO − CCCP])

### 2.16. Measuring Priming in Solid Tumors Using PRIMABs

Immunohistochemistry on formalin-fixed and paraffin-embedded (FFPE) tissue/cell substrates was performed as described above.

### 2.17. Statistical Analysis

Statistical analyses were performed using statistical software programs within Prism (v8.3; GraphPad Software, La Jolla, CA, USA). The measurements made using the PRIMABs were assessed for significance over background. Student’s two-tailed *t*-test was used for the analysis. *p* < 0.05 was considered significant. We used Pearson’s coefficient to determine if cancer cell sensitivity to treatment, in this case IC50s to the BH3 mimetics, would be associated with the binding signals of the PRIMABs. We assumed an R value > 0.5 would indicate significant association. For BH3-profiling in AML samples, biomarkers were analyzed by testing the association between the PRIMAB biomarker status and the MFI from the blast gate or the BH3 profiling assay readout (percentage priming for BIM 0.1 µM or MS1 50 µM in blast gate) and the responder or nonresponder classification. Here, we examined the relationship between BIM priming and complete remission (EFS) in patients treated with a standard-of-care-based 7 + 3 regimen. Univariate comparisons were made using Mann–Whitney test; all reported *p*-values are two-sided. The threshold for significance was *p* < 0.05 for the primary analyses.

## 3. Results

### 3.1. Development of Antibodies Against Conformation Induced Epitopes in Pro-Survival BCL-2 Family Protein: BIM Complexes

**Figure 1:**
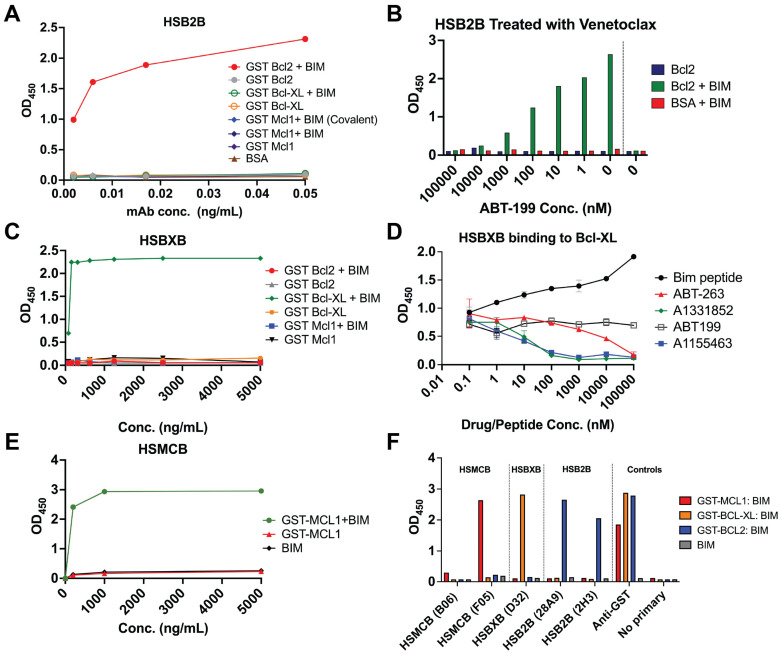
PRIMAB production. (**A**) Supernatants collected from hybridomas from mice immunized with BCL-XL:BIM, BCL-2:BIM, or MCL-1:BIM covalent complexes were screened for selective binding to non-covalent immunogen complexes. ELISA readouts showing selective binding of mAb raised against the BCL-2:BIM covalent complex. Of 950 clones screened, 14 were highly selective; shown here is clone 2H3 that is a Heterodimer specific BCL-2: BIM (HSB2B). HSB2B not interact with BCL-2 or BIM protein alone but does bind to the BCL-2:BIM complex in a concentration-dependent manner. (**B**) The selective BCL-2 BH3-mimetic ABT199 (Venetoclax) was used to disrupt the BCL2:BIM complex. The PRIMAB signal aligned with Venetoclax treatment in a dose-dependent manner as determined by ELISA. (**C**) Supernatant from Hybridoma clone D32 raised against the BCL-XL:BIM complex was applied to the GST-BCL-XL:BIM complex and negative controls as indicated. The HSBXB D32 clone does not bind BCL-XL alone or to the other anti-apoptotic protein:BIM complexes. (**D**) The HSBXB clone was treated with the BCL2 selective BH3-mimetic Venetoclax or with the BCL-XL selective BH3-mimetic A1155463 at the indicated concentrations for 1 h. As indicated by ELISA, the HSBXB is not displaced by Venetoclax but is displaced by A1155463. (**E**) ELISA readouts showing selective binding of mAbs raised against the MCL-1:BIM complex. The HSMCB interacts with the MCL-1:BIM complex but neither protein alone. (**F**) To explore possible crossover binding of the various PRIMABs, biotinylated BIM BH3-domain containing peptides complexed with GST-MCL-1, GST-BCL-2, and GST-BCL-XL were incubated with each PRIMAB. Zero cross reactivity between any one of the PRIMABs and complexes used to make the others was observed.

Mouse mAbs for each complex were produced by generating hybridomas from lymphocytes extracted from mice with high serological IgG titers for the immunogen (as determined by ELISA). These mAbs were designated heterodimer-specific BCL-2:BIM (HSB2B), heterodimer-specific BCL-XL:BIM (HSBXB), and heterodimer-specific MCL-1:BIM (HSMCB). Clones were selected based on specific binding to the pro-survival BCL-2:BIM complex but not monomers of unbound BCL-2, BCL-XL, MCL-1, or BIM (Figure 1A,C,E and Supplemental Figure S1). PRIMAB detection of BCL-2:BIM complex on ELISA plates was blocked in a dose-dependent fashion by the BCL-2 selective BH3 mimetic ABT-199/Venetoclax (Figure 1B). Similarly, the BCL-XL-selective BH3 mimetic, A1155463, but not ABT-199/Venetoclax, blocked the binding of BCL-XL:BIM (HSBXB) (Figure 1D). To demonstrate a further lack of cross-reactivity among HSB2B, HSBXB, and HSMCB, each of the PRIMAB clones was tested against each of the other pro-survival BCL-2:BIM protein complexes by ELISA (Figure 1F). Hence, a panel of three sets of mAbs specific for three priming states consisting of BCL-2 family members in complex with BIM was generated.

### 3.2. PRIMABs Detected Priming PPIs in Cell Lines by Flow Cytometry

**Figure 2:**
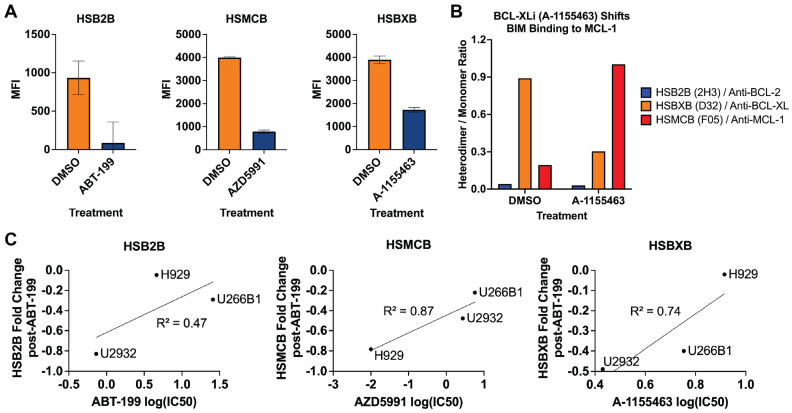
Various BCL-2 PPI disruptions by BH3-mimetics are detected in cells using PRIMBS. (**A**) Cell lines shown to have high levels MCL-1:BIM, BCL-2:BIM, or BCL-XL:BIM complexes were treated with the BH3-mimetics AZD5591, ABT-199, or A115463 respectively. Following treatments cells, were prepared and assessed by flow cytometry for complex signal. Error bars are the standard deviation (SD) for 3 wells. (**B**) Displacement of BIM from BCL-XL is detected by the HXBXB clone D32 in the megakaryoblast cell line SET-2 following treatment with A115463. This disruption of the BCL-XL:BIM complex is accompanied by a compensatory increase in the MCL-1:BIM complex as detected by the HSMCB PRIMAB. (**C**) The IC_50_s of cell lines U2932, H929, and U266B1 were determined for each of the BH3-mimetics ABT-199, AZD5591, and A1155463, respectively. PRIMAB readings were made at baseline and following 1 h of treatment at the determined IC_50_ doses. Baseline readings were set at 1 and the post treatment % fold change in the BCL-2:BIM complex measurements was plotted against the IC_50_. R^2^ values: 0.4, 0.87, 0.74, for HSB2B, HSMCB, HSBXB, respectively, indicate strong dependence of BH3-mimetic sensitivity on priming.

To screen for the likely clinical utility of the PRIMABs in liquid tumors such as AML, it was important to first establish their ability to detect BCL-2 family PPIs in cells in addition to purified protein complexes. This was critical for developing a flow-cytometry-based assay that would allow us to measure mitochondrial priming in blood cancer samples. First, using purified protein complexes and ELISAs, each PRIMAB signal was reduced following treatment of cells with BH3 mimetics established to disrupt the respective complex recognized. We then measured BCL-2 family PPIs in cell lines with established sensitivities to BH3-mimetics specific for BCL-2 (U2932), BCL-XL (U266B1), and MCL-1 (H929) before and after treatments with AZD5991 (targeting MCL-1 complexes), Venetoclax (targeting BCL-2 complexes), and A1155463 (targeting BCL-XL complexes). Corresponding PRIMAB bindings were reduced with the addition of the BH3-mimetic that uncoupled the targeted antigenic complex, confirming accurate PPI detection (Figure 2A). Treatment times of 1 to 6 h and the addition of the caspase inhibitor Z-VAD-FMK allowed detection of the priming shifts as a pre-apoptotic event. Viability was assessed using propidium Iodine (PI) staining and forward scatter (FSC) and away from side scatter (SSC). The total BCL-2, MCL-1, BCL-XL, and BIM expression levels remained constant in these gates during treatment (Supplemental Figures S3–S5).

We next determined whether PRIMABs could detect pharmacodynamic shifts to BCL-2 family PPIs that were not directly targeted. Accordingly, we examined the capability of the PRIMABs to identify such shifts in priming state by measuring levels of pro-survival protein complexes with HSMCB, HSBXB, and HSB2B PRIMABs (as well as constituent proteins) before and after treatment of the MCL-1-primed SET-2 megakaryoblast cell line with A1155463 to specifically target BCL-XL. Here, we readily demonstrated a disruption of the BCL-XL:BIM complex as a reduction in the HSBXB signal relative to the total BCL-XL signal (Figure 2B). This was accompanied by an increase in the HSMCB signal relative to total MCL-1 while the HSB2B signal relative to total BCL-2 remained unaffected. Hence, a shift in priming state from BCL-XL to MCL-1 was identified.

We measured PRIMAB signals early in the mitochondrial signaling pathway following 1 h of BH3 mimetic treatment. Post-treatment, we saw that the extent of the shift in PRIMABs signals (% change from untreated) correlated to the IC_50_s of the PRIMAB matched BH3 mimetic (Figure 2C).

### 3.3. PRIMABs Detected Priming PPIs in Cells by IF and IHC

**Figure 3:**
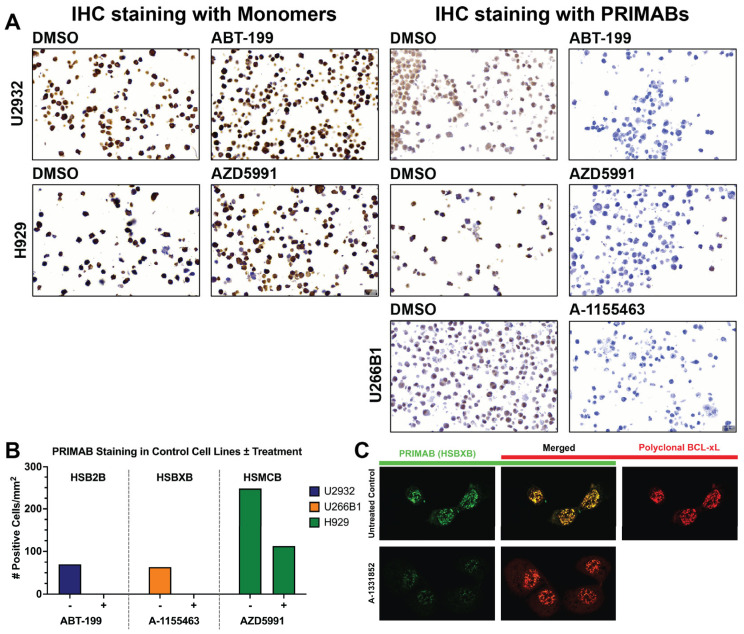
BH3-mimetic shifting of priming complexes in cells can be visualized with PRIMABs. (**A**) Cell blocks were made from ABT-199, A1155463, and AZD5591 treated and untreated U2932, U266B1, and H929 cells to generate TMA slides PRIMABs were used to stain cells as indicated. IHC imaging was performed. Scale bar: 0.020 mm. (**B**) Pixel intensity of IHC in (**A**) was quantified using ImageQuant 800. (**C**) Immunofluorescence imaging of HCC1937 breast cancer cells treated with the BCL-XL BH3-mimetic A1331852 was performed using HSBXB and BCL-XL antibody to measure the BCL-XL:BIM complex and BCL-XL protein alone, respectively. Magnification: 63×. BCL-XL signal (red) colocalizes with HSBXB (green) as indicated in the shift of the green to yellow in untreated cells.

We next established the ability of PRIMABs to detect intact pro-survival BCL-2 protein–BIM complexes using immunofluorescence (IF) and immunohistochemistry (IHC) which would have future applications for determining the priming state of solid cancers. Here, the HCC1937 breast cancer cell line was treated with the BCL-XL:BIM-disrupting BH3-mimetic A1155463 and stained using either the HSBXB PRIMAB or the anti-BCL-XL antibody (Figure 3C). As expected, the complex detected with HSBXB by IF clearly dissipated in the treated cells compared with the untreated cells, while the total BCL-XL signal was unaffected. To extend these data into a formalin-fixed and paraffin-embedded (FFPE) IHC format, untreated MCF-7 cells or those treated with BCL-2-targeting ABT-199/Venetoclax, BCL-XL-targeting A1155463, or MCL-1-targeting AZD5991 were prepared as FFPE slides, and the pro-survival protein–BIM complexes were stained using PRIMABs. In each case, the disruption of the complex was readily detected (Figure 3A,B), while the expression of the individual pro-survival proteins being targeted remained constant (Supplemental Figure S3).

**Figure 4:**
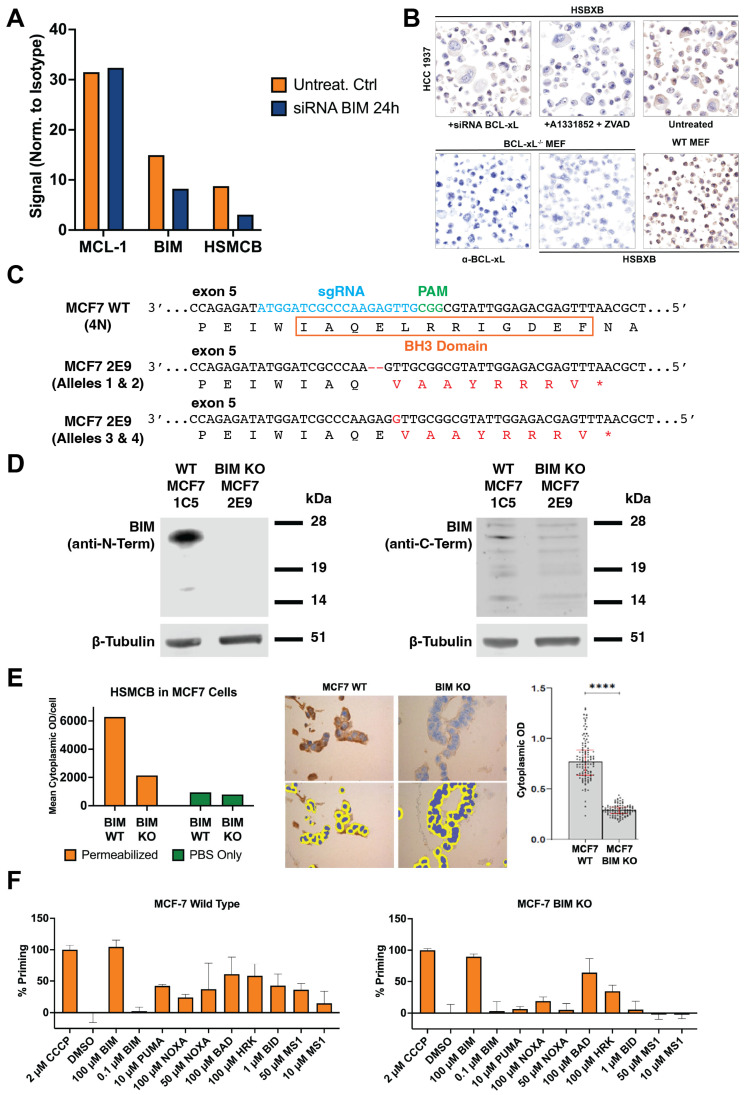
BIM KD/KO impacts PRIMAB staining and priming. (**A**) HCC1937 breast cancer cells were treated with siRNA targeting BIM. Following treatment, intracellular staining of the MCL-1:BIM complex and total MCL-1, including the unbound MCL-1, was performed. Flow cytometry analysis of the cell line samples are reported as MFI. Following treatment, MCL-1 levels remained consistent with levels in untreated cells, while the level of MCL-1:BIM complex was reduced. The level of reduction on the complex coincided with the KD of BIM protein. (**B**) HCC1937 breast cancer cells were treated with BCL-XL targeting siRNA or BCL-XL selective BH3-mimetic A-1331852 and prepared into cell blocks arrayed into TMAs following treatment. IHC was performed and images collected provide a visual assessment of the loss of the BCL-XL:BIM complex measured by HSBXB in the siRNA treated cells at a similar level as the A-1331852 treated cells. In addition, MEF-WT and MEF-BIM ^-/-^ cells were made into cell blocks and arrayed onto TMAs. The BSBXB-PRIMAB signal and the BCL-XL signals were reduced in the MEF-BIM ^-/-^ cells. (**C**) CRISPR/Cas9 was used to target exon 5 of *Bim* in a tetraploid human breast cancer cell line (MCF7). The MCF7 2E9 *BIM* knockout cell line has a two base pair deletion (c.451-452 delGA) in two alleles and a 1 bp insertion (c.452_453 insG) in the other two alleles as confirmed by NGS sequencing. Position of the guide RNA is shown in blue, PAM site in green, and the BH3 domain indicated in orange. (**D**) Western blot analysis showing absence of BIM in MCF7 2E9 compared to otherwise isogenic wild-type MCF7 line (1C5). (**E**) MCF7-WT and MCF7-BIM KO were stained with HSMCB. The flow readout (MFI) indicated a significant loss of signal in the permeabilized BIM KO cells compared to the permeabilized WT cells. The signal in non-permeabilized cells indicated nonspecific background staining. The MCF7-WT and MCF7-BIM KO cells were made into cell blocks and used to generate TMAs. IHC images on samples stained with HSMCB. Images of cytoplasmic segmented staining were analyzed using HALO. Student’s two-tailed *t*-test was used for analysis. Magnification: 20×. (**F**) BH3 profiling was performed on MCF7-WT and MCF7-BIM KO permeabilized and treated with BH3-mimetic peptides and controls as indicated.

We next confirmed in cells that the HSMCB (F05 mAb clone) PRIMAB binding required BIM. To this end, we knocked down BIM in MCF-7 cells using siRNA and measured the HSMCB signal. Using flow cytometry, we saw a reduction in the HSMCB signal (MFI) in the BIM-siRNA-treated cells compared with untreated cells (Figure 4A). The level of MCL-1 protein remained constant in treated and untreated cells. Next, we used IHC to measure the PRIMAB HSBXB signal in BIM-siRNA-treated, BCL-XL–BH3-mimetic-A1331852-treated, and untreated HCC breast cancer cells. We saw reductions in the MCL-1:BIM complex in the BIM-siRNA-treated cells and in the A1331852-treated cells compared with untreated cells (Figure 4B). Using HSBXB IHC, we looked for the BCL-XL:BIM complexes in *Bclxl^-/-^* mouse embryo fibroblast (MEF) cells. First, we confirmed that BCLXL was knocked out, and then we observed an absence of HSBXB signal in the *Bclxl^-/-^* MEFs (Figure 4B). Taken together, these findings indicate that the HSMCB and HSBXB PRIMABs are reliant on BIM.

To obtain a cell line with homogenous loss of BIM, we made BIM knockout (KO) MCF7 cell lines using CRISPR (Figure 4C,D). To show the requirement for BIM binding, we fixed, permeabilized, and stained MCF7 wild type (WT) and BIM KO clone 2E9 cells with HSMCB and found a fourfold reduction in signal in the KO cell by flow cytometry. In the fixed but nonpermeabilized cells, there was a similar, low-level background signal in WT and the KO cells. Given the intracellular location of the BCL-2 family complexes, this was likely nonspecific cell surface staining.

We asked if BIM KO would affect the priming state as measured by a range of BH3 domain peptides using the BH3 profiling method. In this test, the peptides were accessed inside WT and BIM KO MCF7 cells to disrupt existing BCL-2 family PPIs. If MOMP resulted, the priming state could therefore be inferred. We used peptides with varying selectivity for binding to BCL-2, BCL-XL, or MCL-1 and saw that MOMP induced by the MCL-1-targeting BH3 domains from NOXA, PUMA, BID, and MS1 [36] were most affected by loss of BIM, while the non-MCL-1-binding BH3 domains from BAD and HRK were not affected (Figure 4F). This finding indicates the importance of knowing if BIM is bound to MCL-1. It also indicates a distinction between PRIMAB readouts and BH3 profiling readouts.

Combined, these data demonstrate that PRIMABs can be applied using a range of techniques suitable for application on solid and liquid cancers and confirmed the specificity of our PRIMABs in the context of a cellular milieu.

### 3.4. PRIMAB^TM^-DX as a Clinical Biomarker Test

We established that PRIMAB measurements of BCL-2 proteins complexed with BIM tracked with the measurable activity of BH3 mimetics, suggesting potential for utility as pharmacodynamic (PD) biomarkers. We next asked if PRIMAB-enabled measurements might provide meaningful biomarkers for other therapies, as other methods for measuring mitochondrial priming have indicated [12,13,14].

**Figure 5:**
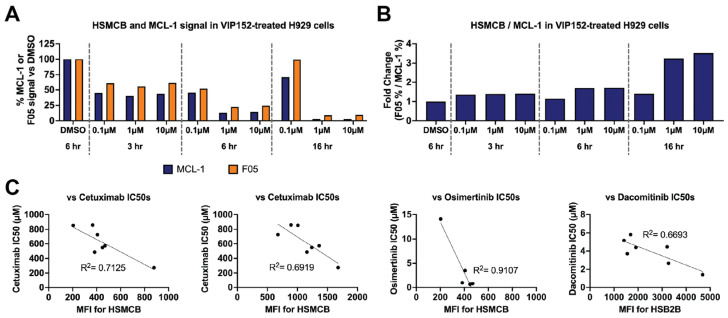
PRIMABs detect CDK9 inhibitor impacting MCL-1:BIM PPIs. (**A**) CDK9 inhibitor VIP152 (Enitociclib) was used to treat H929 cells, and both MCL-1 protein and MCL-1:BIM complex levels were measured by flow cytometry. (**B**) HSMCB signal measured in 5A shifts up in a dose- and time-dependent manner relative to total MCL-1. (**C**) PRIMAB measurements of BCL-2:BIM, BCL-XL:BIM and MCL-1:BIM complexes in human leukemia cell lines KG1a, MV411, HAL929.1.7, EOL-1, SKM-1, F39-P. Molm-13, and NB4 were obtained using Flow cytometry. These measurements were compared to the Broad Institute Cancer Cell Encyclopedia-reported IC_50_s of EGFR inhibitors Osimertinib, Cetuximab, and Dacomitinib for each cell line. HSMCB PRIMAB clones, F05 and B06 respectively, aligned with the reported cell lines IC_50_s for Osimertinib (R^2^ = 0.71, R^2^ = 0.69) and clone F05 with Cetuximab (R^2^ = 0.067), while the PRIMAB readout of HSB2B aligned with the cell lines IC_50_s for Dacomitinib (R^2^ = 0.067).

Initially, we asked if the HSMCB PRIMAB measurements in MCL-1:BIM tracked with H929 cell response to the CDK9 inhibitor Enitociclib, a drug that blocks MCL-1 transcription, reducing MCL-1 protein levels [37]. We showed that treatment with Enitociclib resulted in reductions in both MCL-1 and the MCL-1:BIM complex as measured by HSMCB in a time- and dose-dependent manner (Figure 5A). We also showed that the reduction in MCL-1 signal relative to the HSMCB signal increased with longer treatment times (Figure 5B). This was consistent with prior studies showing that BIM binding stabilizes the MCL-1 protein [28,38].

With this in mind, we looked at MCL-1 or BCL-XL priming related to EGFR therapeutic efficacy, as this priming was reported by others to be significant [39,40]. We did see that HSMCB readouts of two clones, F05 and B06, correlated with IC_50_ values of the EGFR inhibitors Cetuximab and Osimertinib. Similarly, treatment with Dacomitinib was associated with a reduction in the BCL-XL:BIM complex as measured by the HSBXB PRIMAB (Figure 5C). These results indicate potential for using the PRIMABs-DX platform as a predictive test for many indications where EGFR inhibition is a component of treatment regimens and suggest that there will be broad applicability of the platform for use with other survival kinases.

**Scheme 1:**
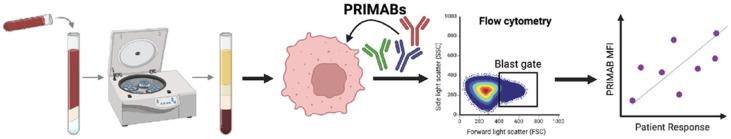
PRIMABs recognized conformational epitopes on intracellular BCL-2 PPIs. Intracellular staining with PRIMABs on Ficoll-purified patient biopsies. Flow cytometry readouts are assessed for correlation to patient response to treatment. Schematic made using BioRender.

**Figure 6:**
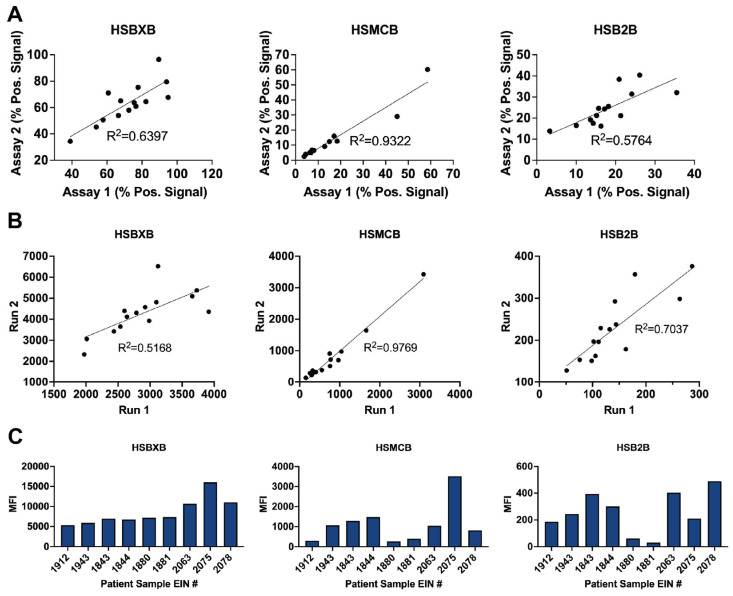
PRIMAB technical performance and signal range in AML patient samples. (**A**) Inter-operator precision for two runs performed by operator 1 (RA) on day 1 and day 2. Data were collected on a panel of human leukemia cell lines (H929, U266B1, U2932, THP1, ML2, KG1a, MV411, HAL929.1.7, EOL-1, SKM-1, F-36P (CVCL_2037), MOLM-13, NB4, and RS4-1). (**B**) Intraoperation accuracy runs were performed by two operators. All assays passed precision (R^2^ > 0.5). (**C**) AML patient samples were fixed, permeabilized, and stained with PRIMABs, then analyzed by flow cytometry to determine the population of complexes present in each patient sample.

Towards developing a clinical test and assessing the range of the PRIMAB signals in patient samples, we analyzed the priming states in 16 AML patient bone marrow (BM) aspirate or peripheral blood (PB) biopsy samples (Scheme 1). Purified leukocyte populations were immunophenotyped for the AML blast cell population and the MFI within both the blast population used for measuring PRIMAB binding. Performance metrics for intra- and interuser accuracy and precision were achieved and provide supporting data for CLIA validation of the PRIMABs-DX, AML-Flow test (Figure 6A). In addition, data from these samples showed a broad range of signals across patients for each PRIMAB, particularly for HSMCB and HSB2B (Figure 6B), which had a similar intensity signal range to the control cell lines (Figure 2A). These data demonstrate the range of heterogeneity of priming states between patients and the plausibility of setting clinically useful positive and negative thresholds for clinical samples.

### 3.5. PRIMAB^TM^-DX Readouts Correlated with Treatment Success in a Retrospective AML Clinical Study

To establish whether PRIMABs could inform treatment responses, AML patient bone marrow and peripheral blood biopsies were collected at time of diagnosis, Ficoll-purified, and viably frozen. Samples were then analyzed with the PRIMABs and with antibodies to each of the constituent anti-apoptotic proteins and BIM. As there were adequate cell counts in these samples, BH3 profiling was also performed. All patients in this cohort reported CRs; therefore, we examined whether PRIMAB responses correlated with event-free survival (EFS).

**Figure 7:**
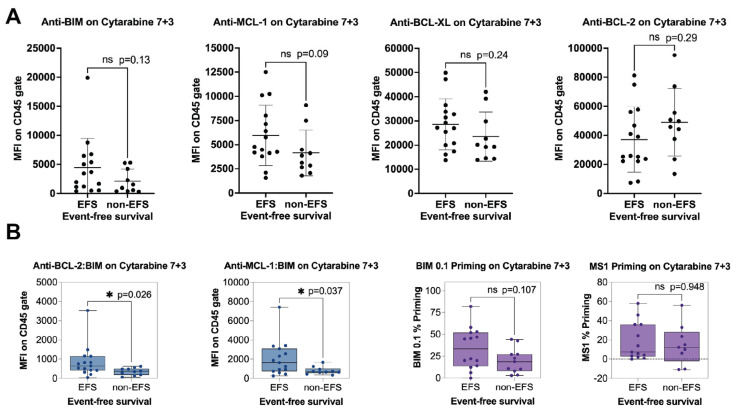
Correlation of PRIMAB signals to AML patient response to treatment. (**A**) Archival AML Patient bone marrow and peripheral blood samples were analyzed with the PRIMABs or with antibodies to the constituent anti-apoptotic proteins and BIM. Testing was performed on ficoll purified peripheral blood biopsies collected immediately prior to treatment with cytarabine based therapy (7+3). These were fixed, permeabilized, and stained with antibodies to BCL-2, BCL-XL, BIM, and MCL-1 and PRIMABs HSB2B, HSMCB, or HSBXB prior to analysis by flow cytometry. Readouts were compared to the event free survival in patients (EFS). Neither the anti-apoptotic proteins nor BIM expression measurements showed significant correlation to EFS. Significant correlation was seen with both the HSB2B PRIMAB (*p* = 0.026) and HSMCB PRIMAB (*p* = 0.037). The HSBXB signal did not correlate to response. (See Supplemental Figure S6). (**B**) BH3 profiling was also performed on the same patient samples. BIM priming did not significantly correlate to EFS (*p* = 0.107), nor did the MS-1 peptide JC1 signal induced by the MS-1 peptide (*p* = 0.958). Student’s two-tailed *t*-test was used for statistical analysis.

For responders, significantly higher MFIs were observed for both the BCL-2:BIM and MCL-1:BIM complex-binding PRIMABs (*p* = 0.026 and 0.037, respectively), but not for the BCL-XL:BIM PRIMAB (Figure S6A). There was no correlation between total BCL-2 family member monomer protein levels and EFS (Figure 7A). This indicates that BCL-2 and MCL-1 priming states, rather than total BCL-2 family protein levels per se, are more relevant for predicting treatment responses. Interestingly, BH3 profiling with the BIM peptide did not show a significant difference (*p* = 0.107) between the EFS and non-EFS cohorts, although there was a trend towards lower priming levels in the non-EFS patients (Figure 7B). This suggests that the PRIMAB approach could have enhanced clinical utility compared with classic BH3 profiling. Further, the results indicate that response and favorable outcomes are significantly associated with the PRIMABs readouts in this initial study. A study empowered to arrive at a validated cut point is underway on a validation set consisting of archival AML patient samples previously treated with a cytarabine-based regimen.

### 3.6. Solid Tumor Applications

**Figure 8:**
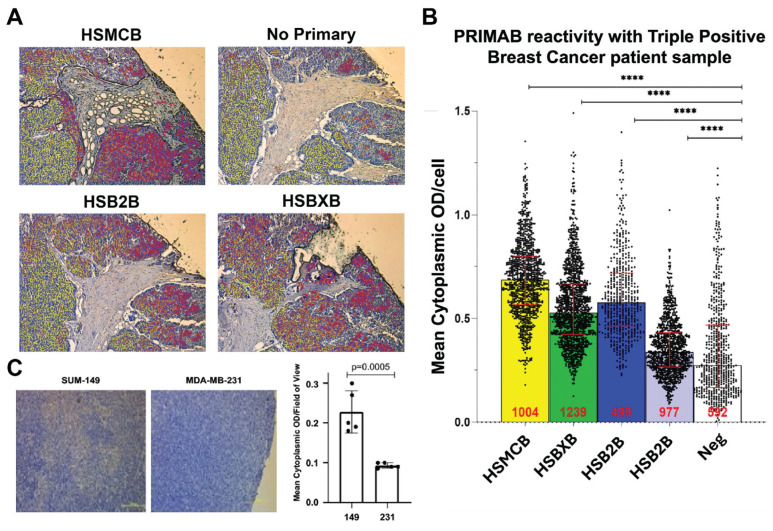
PRIMABs priming measurements in fixed solid tumor samples. (**A**) PRIMABs were used to detect the priming state of small cell lung cancer patient tumor biopsies prepared as FFPE. False color reflects intensity of staining (mean cytoplasmic OD); yellow = low; orange = medium; red = high. (**B**) TMA analysis of triple positive breast cancer patient samples was stained with PRIMABs, and quantification was performed using HALO imaging software. Red numbers within respective bars indicate the number of cells analyzed. The significance of the PRIMAB signals over background is extremely high (*p* < 0.0001). Data indicates that PRIMABs can identify the BCL-2:BIM, BCL-XL:BM, and MCL-1:BIM complexes in both (**A**) small cell lung cancer and (**B**) triple positive breast lung cancer FFPE samples and that they are suitable for use in patient tissue. (**C**) In xenograft mouse models of breast cancer cell lines SUM-149 or MDA MB-231 we show that the MCL-1 inhibitor AZD5991 treatment induced tumor regression in an MCL-1-primed (SUM-149) but not an unprimed (MDA-MB-231) line. MCL-1:BIM priming is detected by HSMCB. Bar chart represents mean cytoplasmic OD for 5 fields of view for the respective stained tissues. The PRIMAB signal to response correlation is highly significant (*p* < 0.0005). Magnification: 10×.

A feature of PRIMABs that distinguishes their malleability to other methods of measuring priming is their ability to be used on nonviable solid tissue cancer samples by IHC, IF, and imaging mass cytometry (IMC). This offers a distinct opportunity to gain insights into priming in solid tumors whilst also collecting granular data on the proximal and distal tumor microenvironment, including data on different immunological cell types and states. As seen, PRIMABs provide robust signals in cell lines prepared for imaging (Figure 2E, Figure 3A,C and Figure 4B,E). We examined IHC images collected on small cell lung cancer tissue sections (Figure 8A). This unique ability to detect priming in solid tumor biopsies may provide an opportunity to add value to widely used IHC biomarkers that (when combined with a PRIMAB readout) could provide enhanced prognostic and predictive utility to solid tumor cancer diagnosis and treatment. As an initial indication of potential predictive utility of the PRIMAB readouts, we showed a distinct staining in xenografted human breast cancer cells that are responsive to MCL-1 inhibitor, AZD5591, or not responsive to that treatment (Figure 8C). There was a clear cutoff in the HSMCB signal strength distinguishing responder from nonresponder cells. Taken together, these data support the promise of PRIMABs as being useful for guiding the use of an MCL-1 targeting BH3-mimetic and most likely other BCL-2 family PPI-disrupting therapies in solid tumors [20].

## 4. Discussion

We set out to build a predictive biomarker test based on the understanding that BCL2 family protein–protein complexes are key determinants of cancer cell survival and cancer cell death. To do this, we devised and built the PRIMABs-DX platform to selectively measure BCL-2 family complexes that indicate the cancer cell priming state. Priming refers to sequestration of the BIM BH3 domain in the BH3 binding pocket of the anti-apoptotic proteins. Its displacement frees it to activate executioner BCL-2 family members BAX and BAK [38]. Realizing that priming state indicates a cancer’s dependency on one or another of the BCL-2 proteins for their survival, this information can be exploited therapeutically.

The approval and use of the BCL-2:BIM complex-disrupting Venetoclax has heightened interest in finding precision medicine tools that could measure priming and, in doing this, identify cancer cells dependencies as vulnerabilities that could be addressed with therapy. Current methods to measure priming include BH3 profiling and measurement of PPIs by pull-down followed by high-resolution ELISA. Though important research tools, these methods have not had significant uptake in the clinical setting, which has left an outstanding need unmet.

The PRIMAB platform relies on detecting epitopes that are present only in heterodimer-complexes and making PRIMABs against them. The PRIMAB targets consist of each of the primary anti-apoptotic proteins BCL-2, MCL-1, and BCL-XL dimerized with the BH3 domain of the pro-apoptotic protein BIM. This BH3-only protein was chosen over all other pro-apoptotic BCL-2 proteins for several reasons, including extensive biochemical and structural analyses in dozens of papers that show that BIM binds all pro-survival BCL-2 proteins with equivalent, high affinity and near-identical binding modes [20,37,41]. In addition, it can directly activate BAX and BAK and is considered the most potent pro-apoptotic BH3-only BCL-2 family member. The low nanomolar binding affinity of the BIM BH3 domain to each of the chosen anti-apoptotic proteins assisted us in making covalent heterodimer complexes that were used as immunogens. Following covalent coupling, purification, and assessment in binding assays, the complexes were used to elicit an immune response, and mAbs to specific heterodimers were selected. The key feature of PRIMAB binding is its requirement for BIM binding or BIM-BH3 peptide binding to the pro-survival protein. We demonstrated this biochemically, using purified protein, and in cells; PRIMABs recognized only intact complexes (Figure 1). No signal was detected when BIM was deleted (Figure 4) or complexes were disrupted with BH3-mimetics (Figure 3).

The capability of PRIMABs to selectively track the occurrence of BH3-domain-induced epitopes make them likely pharmacodynamic biomarkers with potential to predict patient response to BH3 mimetic therapies. It is held that cancer cell adaptations to BH3-mimetic treatment can involve a shift in the priming status of tumor cells. For example, high BCL-2:BIM priming generally indicates responsiveness to BCL-2-targeting BH3-mimetics. However, if cancer cells are MCL-1 primed, to a greater or lesser degree, then the patients harboring those “primed to adapt” cancer cells might be steered to a treatment that could target the MCL-1:BIM complexes cells even before relapse from BCL-2:BIM targeting treatment. In essence, treatment-induced priming can shift from BCL-2 to MCL-1 and is associated with resistance to Venetoclax [9,42,43] and other therapies that target the intrinsic apoptotic pathway directly or indirectly [44,45,46,47]. Figure 2B shows that PRIMABs can detect such a shift, indicating potential for them being used as resistance biomarkers for BH3 mimetics therapies.

Importantly, PRIMAB signals are observed in a cellular milieu, essential for clinical application (Figure 3 and Figure 8). An important and distinct feature of the PRIMAB platform is utility in visualizing cancer cell priming enabling visual tracking of drug-induced priming shifts in cells, using microscopy and IHC for that capability (Figure 3 and Figure 8). PRIMABs can identify vulnerabilities of subsets of cells in the context of the tumor microenvironment, adding an important dimension to the data. Additionally, PRIMABs can enable intracellular visualization of BCL-2 family complexes relative to the unbound complex constituent proteins (Figure 3C). These capabilities are unique compared with other protein–protein interaction platforms and will enable quick uptake of the PRIMABs for use in solid tumors.

An important drug class for solid tumors are the EGFR inhibitors. Mitochondrial priming is a key indicator of cancer cell response to EGFR inhibition [46,47] as it is with other survival kinase inhibitors used in treating solid tumors [20,46,47]. While BH3 profiling and pull-down approaches can be applied to platforms for solid tumors, these approaches are limited by assay complexity and difficulty in obtaining homogeneous patient cancer cell preparations [12,13,14]. PRIMABs overcome these issues, showing promise in solid tumor applications and are currently being developed as IHC biomarker tests. Here, we showed that HSMCB readout correlated to EGFR cell response to the inhibitors Cetuximab and Osimertinib. Similarly, treatment with Dacomitinib correlated to HSB2B signal (Figure 5C). We also showed successful HSMCB PRIMAB tracking with CDK-9 inhibitor (Figure 5A) and with MCL-1 mimetic in breast cancer xenograft (Figure 8C). These results indicate potential for using the PRIMABs-DX platform as a predictive test for many indications where EGFR inhibition is a component of treatment regimens and suggest that there will be broad applicability of the platform for use with cell cycle inhibitors, including CDK9 inhibitors, and a range of apoptosis-inducing survival kinase inhibitors.

A primary motivation in developing PRIMABs is to provide them as tools for drug development and for clinical research and clinical care communities. To this end, PRIMABs are currently undergoing CLIA validation for use in AML. The data reported here demonstrated the range of signals achievable in a cohort of naive AML patient samples purchased from commercial vendors. Expanded studies have also demonstrated intraday, interday, intrauser, and interuser accuracy, precision, and reproducibility according to CLIA protocols and accomplished analytical validation (Figure 6). Future studies will progress this work to clinical validation in archival or ongoing clinical trials.

During the past 15 or so years, BH3 profiling has contributed significantly to the understanding of mitochondrial priming and cancer cell survival dependencies. It has been used by many labs in a large range of clinical cancer studies, resulting in more than one hundred published papers. To help us confirm the potential clinical utility of PRIMABs, we benchmarked them against BH3 profiling in archived clinical blast samples collected from newly diagnosed AML patients who had been treated with standard-of-care (7 + 3 cytarabine + anthracycline). The readouts from the BH3 profiling were from BH3 peptides, or adjusted peptide readouts, that indicated priming from BCL-2, BCL-XL, or MCL-1. When compared with event-free survival, there was not significant correlation to any of the peptides or peptide readouts. In contrast, the HSMCB and HSB2B PRIMABs showed significant correlation to the event-free survival, while antibodies to unbound MCL-1, BCL-2, BCL-XL, or BIM proteins did not show significant correlations. While the study size was small, this does indicate a trend that we will follow with a better-powered sample size. However, it should be noted that while these two platforms measure a similar feature in cancer cells, they are completely distinct. BH3 profiling relies on a functional response to exogenous reagents over a time course, while PRIMABs provide a snapshot of the critical protein complexes that determine the priming state. The direct comparisons of these approaches reflect the fundamental differences between the assays. Reflecting this, comparisons between them are variable (Figure 6 and Supplemental Figure S7). Nonetheless, in this study, the PRIMAB readouts showed significant correlation to AML patient event-free survival to standard of care.

The PRIMABs-DX platform provides a novel approach for measuring BCL-2 family PPIs involved in cancer cell chemosensitivity and resistance, with the potential to complement widely applied genetic testing. Unlike other, highly complex methods that directly or indirectly measure apoptosis-regulating PPIs, our approach is suitable for clinical use, as it employs antibodies that can be utilized in fixed biopsied tissue using techniques such as immunohistochemistry and flow cytometry that are widely used in pathology labs worldwide. To our knowledge, similar antibodies capable of detecting pro-survival–pro-apoptotic protein complexes that dictate the priming state of a cell have never been reported in the literature. Importantly, the specificity of our antibodies to the three major BCL-2 family heterodimer protein complexes (i.e., BCL-2, MCL-1, and BCL-XL with BIM) that dictate cancer cell priming was validated here using both purified proteins and cells, including knockdown/knockout lines. We also showed that PRIMABs identify dynamic shifts in the priming complexes. The HSBXB PRIMAB identified a high level of the BCL-XL:BIM complex in cells before treatment and a reduced signal upon treatment with a BCL-XL BH3 mimetic (A1155463). The HSMCB PRIMAB identified a commensurate increase in the MCL-1:BIM complex. This ability to measure the BH3-mimetic target complex and its dissolution following treatment of priming complexes represents a powerful pharmacodynamic tool as well as a clinically relevant readout for identifying and tracking cancer cell adaptations to treatment with BH3 mimetics or treatment with a broad array of apoptosis inducing therapeutics (Figure 2B). This is an important capability, as there is strong concern about patient resistance to Venetoclax that is thought to be mediated by changes in cancer cells’ dependencies from one BCL-2 family protein to another, for instance, from BCL2 priming to MCL1 priming. Knowing this allows adjusting treatment to target the appropriate vulnerability [26,27]. Such a shift in the priming state has been shown to underlie a BH3-mimetic resistance mechanism [8,9,10,11,12]. Hence, PRIMABs could provide useful tools to identify such treatment blocks.

Several published studies have used BH3 profiling and other methods to show the clinical importance of measuring mitochondrial priming. However, while these assays provide important and useful information, none have achieved significant uptake in the clinical setting. The PRIMAB DX approach offers advantages that make it amenable to clinical use. The requirements of tissue sample viability and processing steps, protein stability in complex biochemical testing, difficulty in controlling cell permeabilization as required for accessing peptides into cells, and others are overcome by using simpler methods. The platform also will provide meaningful metrics for complementing other approaches that measure treatment response in ex vivo biopsied tumor cells, potentially helping overcome complications due to tumor tissue heterogeneity and the requirement to enzymatically or mechanically disperse cells prior to treatment. Approaches that measure protein complexes using pull-down ELISA are accurate but require complex purification of intended cell populations. This is not the case with PRIMABs, where tissue sections, including tumor microenvironment, can be assessed at essentially a single-cell level. PRIMAB signals in tumor tissue FFPE show spatially distinct information and cell type location. This capability will contribute to tracking cell subpopulations’ function in the context of the tumor microenvironment.

As discussed above, PRIMABs can predict patient responses to cytarabine-based treatment of AML that were not detected as being significant by BH3 profiling. Therefore, while BH3 profiling has been shown to provide clinically relevant insights, PRIMABs potentially have additional benefits and predictive power for clinical applications.

One important consideration for our platform is how mutations and post-translational modification could influence epitopes associated with PRIMAB binding. For example, the epitopes of the PRIMABs have yet to be mapped, so it is still to be determined whether unreported post-translational modifications affect PRIMAB signals beyond those due to BH3-induced conformational changes. Additionally, some mutations that emerge following Venetoclax treatment and drive resistance [39,40] are located close to the BH3 binding groove and therefore could be associated with the PRIMAB epitopes. Whether such mutations influence binding remains unknown, and though they could potentially influence the degree of priming we measure in some contexts, our present data show robust PRIMAB signals across cell lines and cancer types, suggesting this may not be a significant issue. Nevertheless, our future studies will endeavor to characterize if such mutations influence our measurements, and to what extent.

PRIMABs have unique potential to provide insights that can complement measurements of mutational and expression level variation features of tumor cells as well as proliferation and effector function of immune cells. With BH3 mimetics that target BCL-2 becoming widely used and those targeting the other anti-apoptotic BCL2 proteins in development, these insights are becoming more important. Clinicians face challenges in learning how to best use these new and emerging drugs, which will be important in both blood and solid tumors. PRIMABs could help define the path to attain broad clinical success for a range of new therapeutic regimens.

## 5. Conclusions

We describe a new diagnostics platform based on a novel panel of antibody-based reagents that have significant research utility and potential clinical utility for the prediction of drug treatment responses or in diagnosing mechanisms underlying treatment resistance in both solid and blood cancers. There is a well-established scientific hypothesis regarding the significance of mitochondrial priming as a functional predictor of cancer cell response to drug-induced apoptosis signaling. The PRIMABs allow us to expand that understanding and bring it into the clinical setting by readily monitoring priming before and during treatment to give a dynamic picture with a simple, reliable method. The data indicate that the PRIMABs biomarker approach provides data to accurately predict patient response. A wider range of clinical samples and trial data will enable further validation for future implementation of the PRIMABs-DX platform.

## Figures and Tables

**Table 1:** Measuring heterodimer complexes using FACS analysis.

IF Labeling for PRIMABs				IF Labeling for Total BCL-2 Multiplexed Proteins				
Antibody	HSB2B	HSBXB	HSMCB	BCL-2	BCL-XL	MCL-1	BIM	CD45
**Species**	Mouse	Mouse	Mouse	Rabbit	Rabbit	Rabbit	Rabbit	Mouse
**Tag**	α-mouse	α-mouse	α-mouse	Direct Tag	Direct Tag	Direct Tag	Direct Tag	Direct Tag
**Fluor**	647 nm (APC Red)	647 nm (APC Red)	647 nm (APC Red)	488 nm (A-488)	647 nm (APC Red)	780 nm (PE-CY7)	575 nm (PE)	450 nm (V450)
**Conc.**	1 µg/mL	1 µg/mL	1 µg/mL	1 µg/mL	1 µg/mL	1 µg/mL	1 µg/mL	0.5 µg/mL
**Vendor**	LifeTechnologies	LifeTechnologies	LifeTechnologies	CST	CST	CST	CST	BD

## Data Availability

Data are available from the corresponding author upon reasonable request.

## Supplementary Materials

The following supporting information can be downloaded at: https://www.mdpi.com/article/10.3390/cancers17111852/s1, Illustration S1: is a schematic illustration depicting the coupling of photoactivatable benzoyl phenylalanine modified BIM-BH3 peptide. Figure S1: Determining selectivity of HSMCB hybridoma clones; Figure S2: Flow cytometry measurements of BCL-2 proteins and BCL-2:BIM complexes in control cell lines; Figure S3: Cell viability in treatment of control cells; Figure S4: BCL-2 protein expression levels in control cells during treatment with BH3 mimetics; Figure S5: ELISA signal of HSMCB Binding MCL-1:BIM complex is disrupted by AZD5991; Figure S6: BCL-XL: BIM PRIMAB (HSBXB) showed no correlation to Event Free Survival (EFS) in AML biopsies from patients treated with Cytarabine Based therapy (7+3); Figure S7: Comparison of PRIMAB (HSBXB) readout and BH3 profiling readout of BCL-XL selective BH3 peptide HRK in leukemia cells; Table S1: Control cell lines test positive and negative for PRIMABs; Table S2: siRNA transfection conditions based on culture dish size; Table S3: Post-fixation optimization for IHC.

## Abbreviations

The following abbreviations are used in this manuscript:

AML	Acute myeloid leukemia
ATCC	American Type Culture Collection
BCL-2	B-cell lymphoma 2
BH3	BCL-2 homology region 3
BM	Bone marrow
BPA	4-benzoyl phenylalanine
CLL	Chronic lymphocytic leukemia
CR	Complete response
EFS	Event-free survival
FBS	Fetal bovine serum
FFPE	Formalin-fixed and paraffin-embedded
GST	Glutathione-S-transferase
HMA	Hypomethylating agents
HSB2B	Heterodimer-specific BCL-2:BIM
HSBXB	Heterodimer-specific BCL-XL:BIM
HSMCB	Heterodimer-specific MCL-1:BIM
IF	Immunofluorescence
IHC	Immunocytochemistry
IMC	Imaging mass cytometry
LDAC	Low-dose cytarabine
mAb	Monoclonal antibody
MCOD	Mean cytoplasmic optical density
MFI	Mean fluorescence intensities
MM	Multiple myeloma
MOMP	Mitochondrial outer membrane permeabilization
NGS	Next-generation sequencing
NR	No response
PB	Peripheral blood
PBS	Phosphate buffered saline
PD	Pharmacodynamic
PEI	Polyethylenimine
PPI	Protein–protein interaction
TMA	Tissue microarray
*v*/*v*	Volume-to-volume
*w*/*v*	Weight-to-volume
