# Supplementary material for: Development of a Novel Biomarker Platform for Profiling Key Protein–Protein Interactions to Predict the Efficacy of BH3-Mimetic Drugs
Source: Cancers (Basel). 2025 May 31;17(11):1852. doi: 10.3390/cancers17111852 (PMC12153628; doi:10.3390/cancers17111852)
Supplement: Supplementary file 1 [file cancers-17-01852-s001.zip › cancers-3611521-SI.pdf]

Supplemental Illustrations & Figures

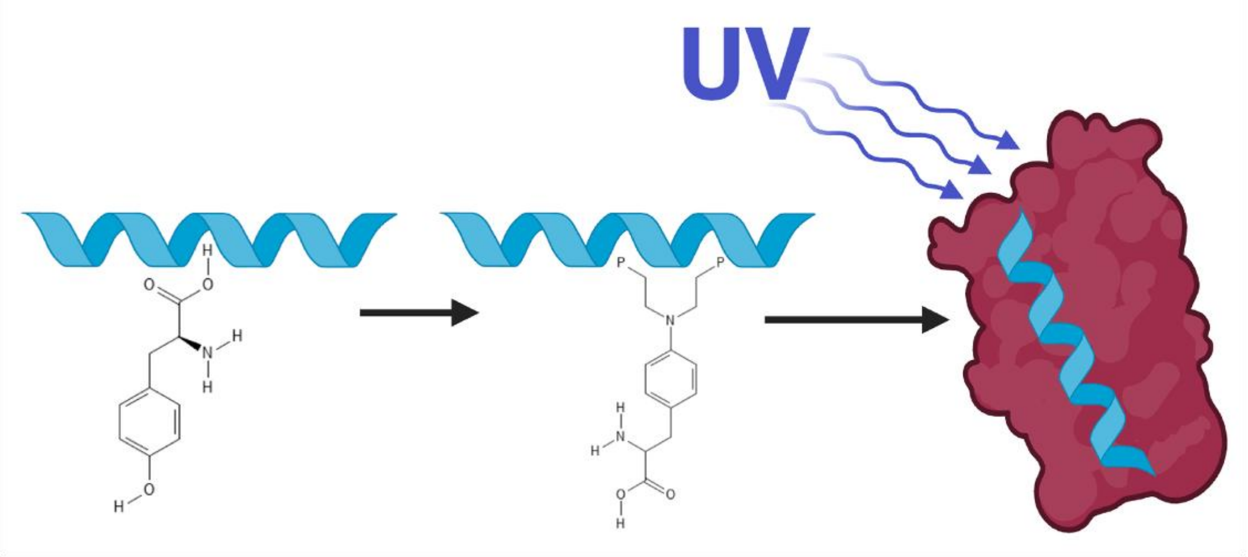

Supplemental Illustration S1: is a schematic illustration depicting the coupling of photoactivatable benzoyl phenylalanine modified BIM-BH3 peptide. This peptide is allowed to be complexed with GST-BCL-XL while being exposed to UV light [35]. The covalent heterodimer was purified on a glutathione-sepharose column and assessed for function before being used to immunize mice. Illustration made using BioRender.

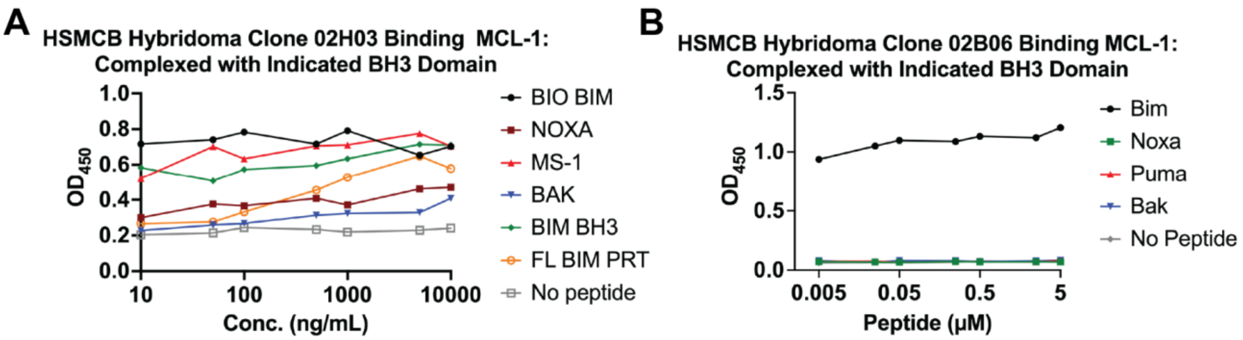

Supplemental Figure S1: Determining selectivity of HSMCB hybridoma clones. A) HSMCB hybridoma clone 02H03 raised against MCL-1:BIM complex and screened on ELISA-glutathione plates coated with GST-MCL-1 alone or complexed with different BH3 domain containing peptides from pro-apoptotic BIM, Noxa, BAK, N-terminal biotinylated BIM, full length BIM protein, and the MS1-BH3 domain. FL BIM PRT stands for full-length BIM protein. **B)** ELISA wells coated with GST-MCL-1 bound only when complexed with BIM-BH3 domain-containing peptides.

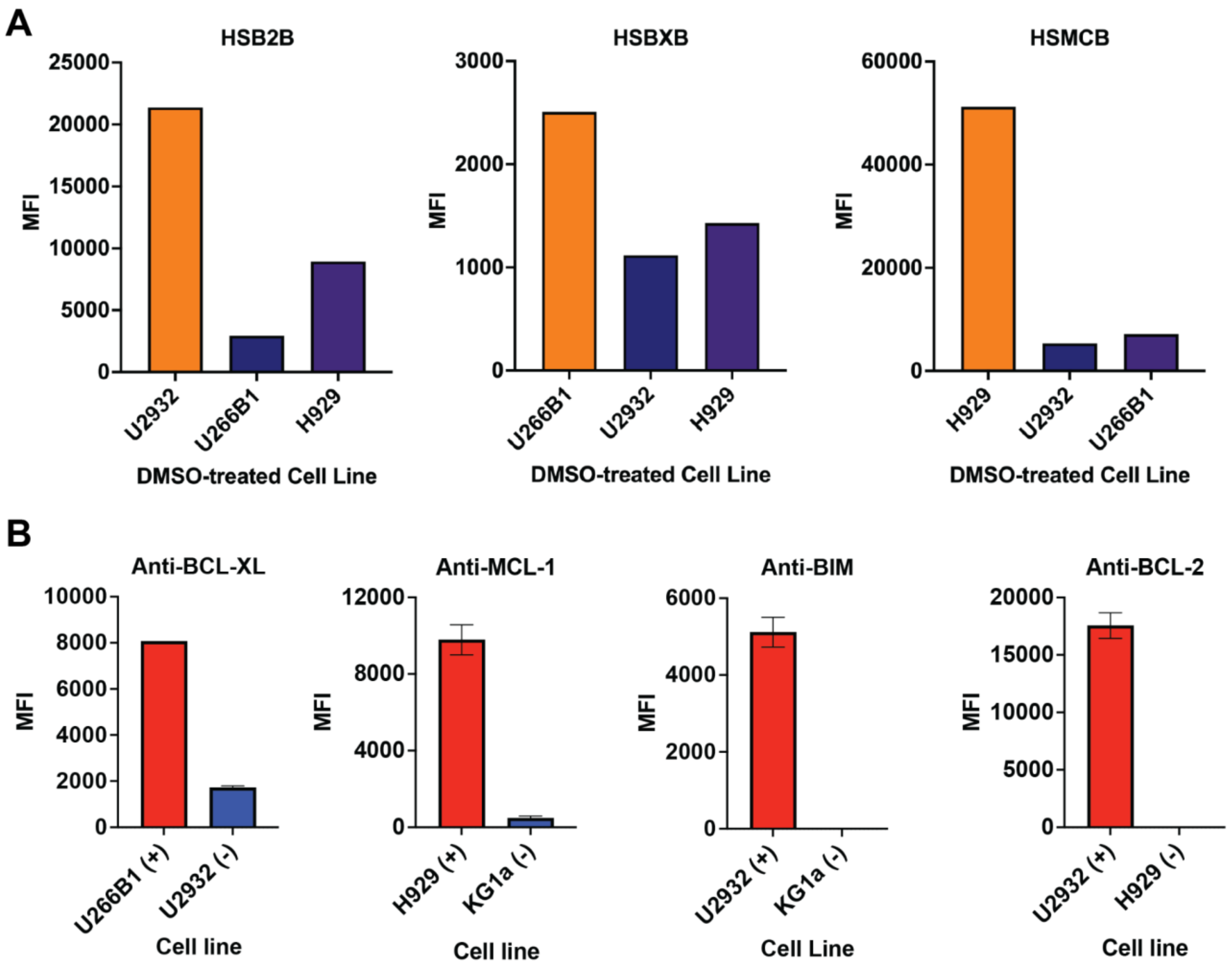

Supplemental Figure S2: Flow cytometry measurements of BCL-2 proteins and BCL-2:BIM complexes in control cell lines. A) Flow cytometry measurements of BCL-2 family complexes carried out in control cell lines U2932, U266B1, and H929 demonstrate specificity of PRIMABs. B) Total amounts of BCL-2 family proteins were analyzed by Flow cytometry in U266B1, U2932, H929, and KG1a control cell lines.

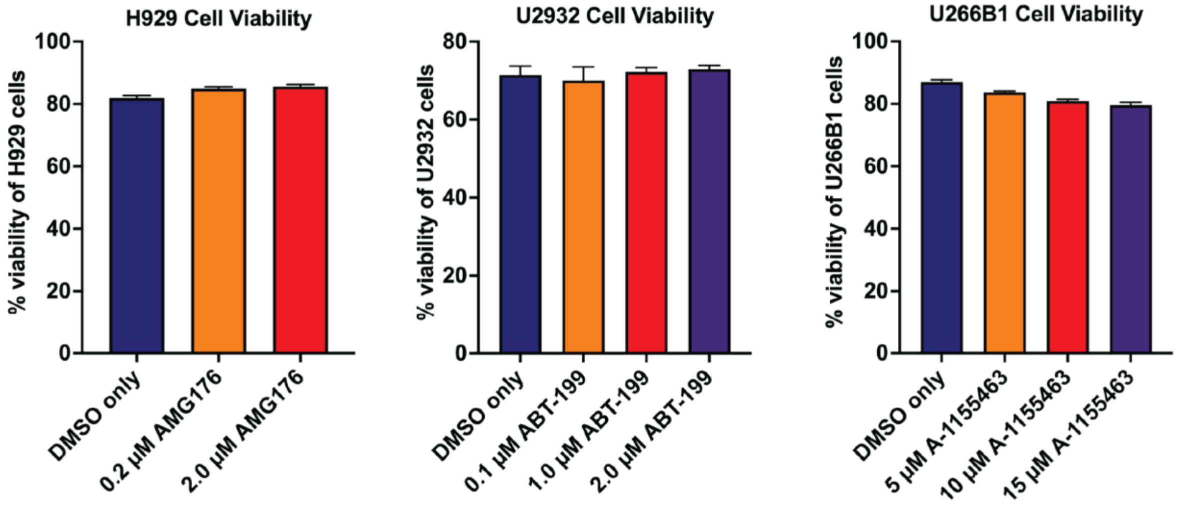

Supplemental Figure S3: Cell viability in treatment of control cells. Flow cytometry with PI staining as an indicator of viability during treatments in control cells H929, U2932, and U266B1 was carried out following 6-hour treatment with the indicated BH3-mimetics plus Z-VAD-FMK caspase inhibitor prior to analysis.

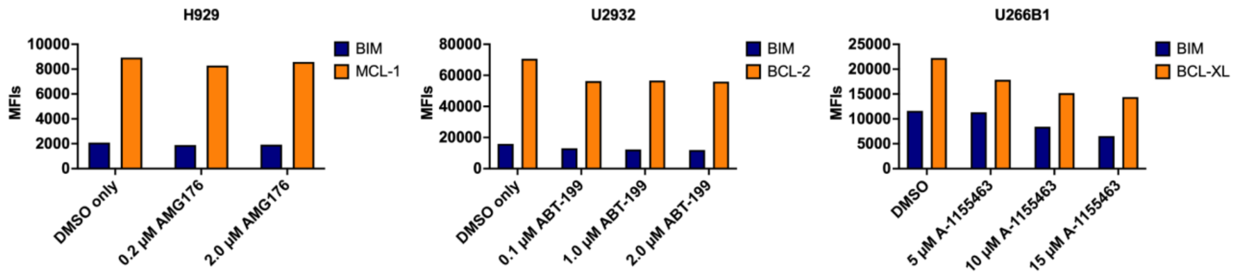

Supplemental Figure S4: BCL-2 protein expression levels in control cells during treatment with BH3 mimetics. BCL-2 family protein expression levels were measured using flow cytometry in control cell lines H929, U2932, and U266B1. Cells were treated with indicated doses of BH3-mimetics ABT199, A1155463, and AMG176; with 50 µM Z-VAD-FMK for 6 hours.

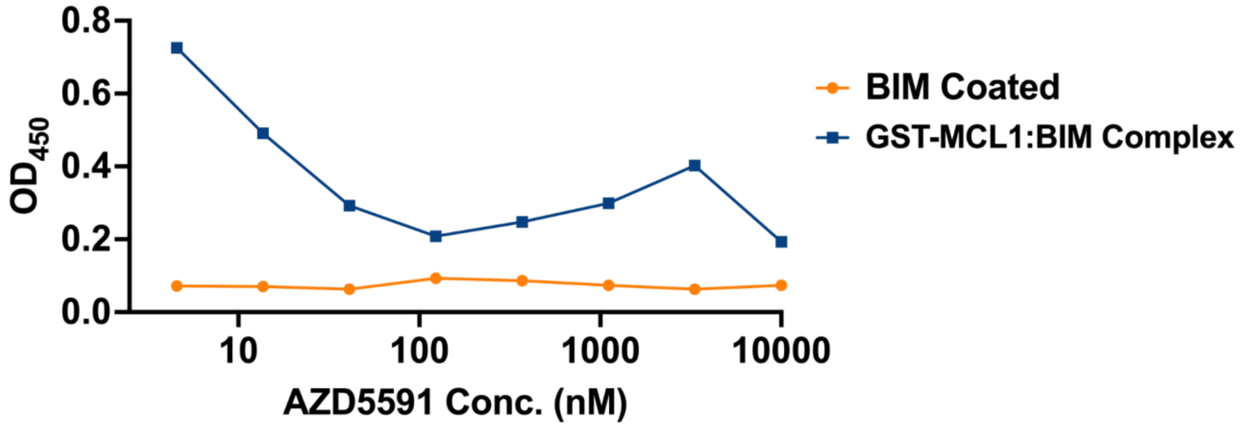

Supplemental Figure S5: ELISA signal of HSMCB Binding MCL-1:BIM complex is disrupted by AZD5991. ELISA measurements of HSMCB binding to GST-MCL-1:BIM complexes followed treatment of the MCL-1:BIM complex with AD5591. Treatments were for 1 hour at room temperature.

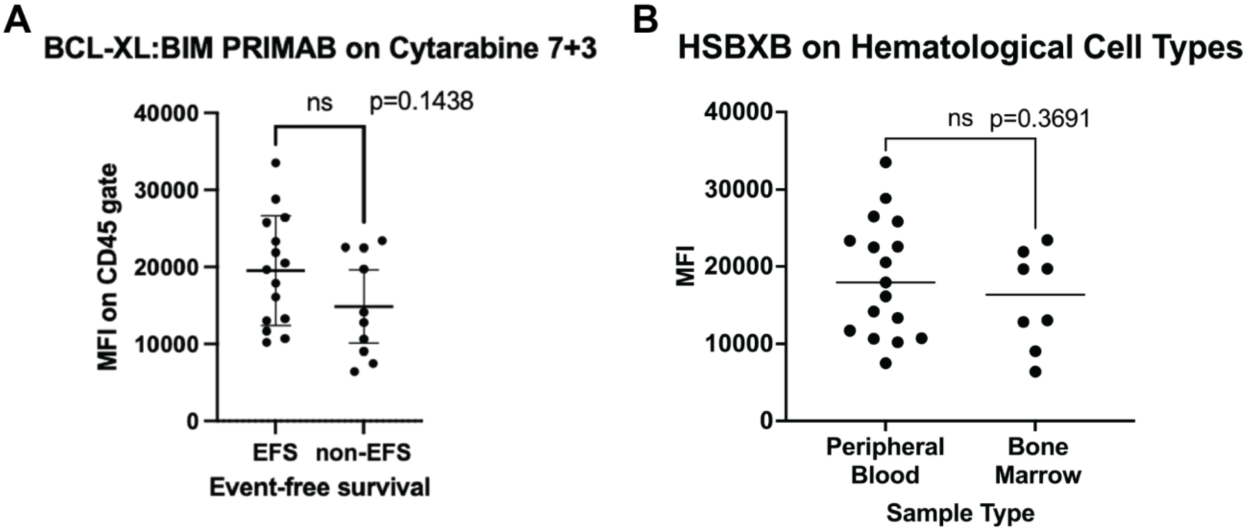

Supplemental Figure S6: BCL-XL : BIM PRIMAB (HSBXB) showed no correlation to Event Free Survival (EFS) in AML biopsies from patients treated with Cytarabine Based therapy (7+3) A) AML patient bone marrow and peripheral blood samples were analyzed with HSBXB. Testing was performed on ficoll-purified peripheral blood and bone marrow biopsies collected immediately prior to treatment with cytarabine based therapy (7+3). Readouts were compared to the event of free survival in patients (EFS). The HSBXB signal did not correlate to EFS. **B)** The HSBXB signal was comparable in bone marrow and peripheral blood from 7+3 treated AML EFS patients.

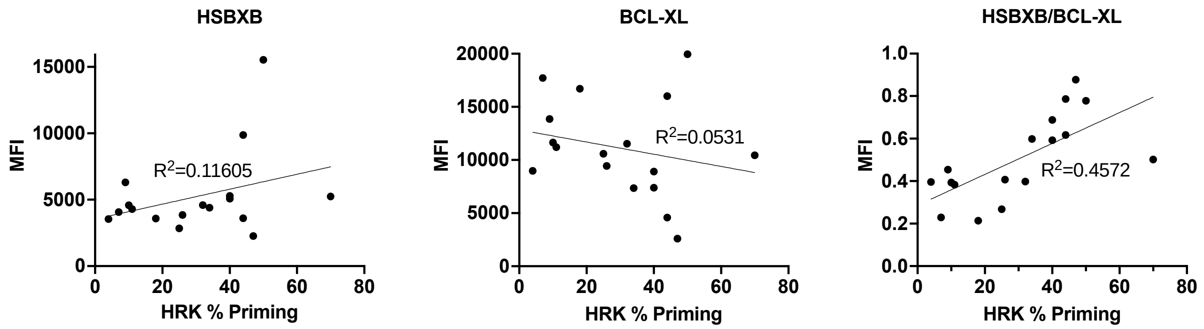

Supplemental Figure S7: Comparison of PRIMAB (HSBXB) readout and BH3 profiling readout of BCL-XL selective BH3 peptide HRK in leukemia cells. Ficoll purified and viably frozen CLL patient peripheral blood specimens were prepared for BH3 profiling, and a small portion of the sample was fixed and stained with HSBXB or with anti-BCL-XL. These samples were analyzed on the FacsCanto 2 flow cytometer. Flow cytometry BH3 profiling was performed. Readouts are Mean Fluorescence Intensity (MFI) within the CD20 gate. The HRK peptide signal is represented as % of the CCCP (uncoupling positive control=100%). HRK % positive is compared to HSBXB staining correlation is Weak (R^2=0.11605); HRK % signal is compared to BCL-XL staining correlation is None (R^2=0.0531); HRK % signal is compared to the HSBXB/BCL-XL correlation is Weak to Moderate (R^2=0.4572).

Supplemental Tables

Supplemental Table S1. Control cell lines test positive and negative for PRIMABs.

PRIMAB	Control	Cell Line	Dependency	Treatment	Drug Target
HSB2B	anti-BCL2	U2932	BCL-2	DMSO	–
				ABT-199	BCL2:BIM
HSMCB	anti-MCL1	H929	MCL-1	DMSO	–
				AZD5991	MCL1:BIM
HSBXB	anti-BCLXL	U266B1	BCL-XL	DMSO	–
				S463	BCLXL:BIM

Supplemental Table S2. siRNA transfection conditions based on culture dish size.

Culture Dish	Surface (mm)	Tube 1 (μL)		Tube 2 (μL)		Growth Medium (mL)
		siRNA (5 μM)	OptiMEM	DharmaFECT	OptiMEM	
6-well	962	10	190	10	190	2
T75	7500	100	1900	100	1900	20
T160	16000	200 (120)	3800 (2280)	200 (120)	3800 (2280)	16 mL or 24 mL per flask

Supplemental Table S3. Post-fixation optimization for IHC.

**Protocol Step**	**Suggested New Reagent**
Antigen Retrieval (pH 9.0)	Antigen Unmasking Solution, Tris-Based (Vector Laboratories, H-3301-250)
Inactivation of Endogenous Peroxidases	BLOXALL Endogenous Blocking Solution, Peroxidase and Alkaline Phosphatase (Vector Laboratories, SP-6000-100)
Secondary Antibody (anti-Mouse)	Peroxidase AffiniPure $F(ab')_2$ Fragment Donkey anti-Mouse IgG (H+L) (Jackson ImmunoResearch Laboratories, 715-036-150)
Secondary Antibody (anti-Rabbit)	Peroxidase AffiniPure $F(ab')_2$ Fragment Donkey anti-Rabbit IgG (H+L) (Jackson ImmunoResearch Laboratories, 711-036-152)
Signal Development	ImmPACT DAB EqV Substrate Kit,Peroxidase (HRP) (Vector Laboratories, #SK-4103)
